# Microbial signals in primary and metastatic brain tumors

**DOI:** 10.1038/s41591-025-03957-4

**Published:** 2025-11-14

**Authors:** Golnaz Morad, Ashish V. Damania, Brenda Melendez, Bharat B. Singh, Fabiana J. Veguilla, Rebecca A. Soto, Yasmine M. Hoballah, Pranoti V. Sahasrabhojane, Matthew C. Wong, Mona M. Ahmed, Rene N. Rico, Kaitlyn N. Lewis, Khalida Wani, Diana D. Shamsutdinova, Rossana N. Lazcano Segura, Davis R. Ingram, Eric A. Goethe, Abderrahman Day, Ivonne I. Flores, Lauren K. McDaniel, Manoj Chelvanambi, Sarah B. Johnson, Florentia Dimitriou, Pravesh Gupta, Shivangi Oberai, M. Anna Zal, Phoebe Doss, Mohamed A. Jamal, Eiko Hayase, Chetna Wathoo, Lisa M. Norberg, Stephanie L. Jenkins, Sara Nass, Joy Gumin, Lihong Long, Jing Yang, Gina R. Bradley, Mahesh Prasad Bekal, Antonio G. Dono, Pavel S. Pichardo-Rojas, Samuel W. Andrewes, Leomar Y. Ballester, Jillian S. Losh, Jiyong Liang, Longfei Huo, Douglas C. Nielsen, Brittany C. Parker Kerrigan, Priscilla K. Brastianos, Natalie Wall Fowlkes, Chia-Chi Chang, Robert R. Jenq, Candelaria Gomez-Manzano, Jason T. Huse, Michael A. Davies, Alexander J. Lazar, Krishna P. Bhat, Nitin Tandon, Yoshua Esquenazi, Christine B. Peterson, Vinay K. Puduvalli, Frederick F. Lang, Christopher D. Johnston, Susan Bullman, Nadim J. Ajami, Sherise D. Ferguson, Jennifer A. Wargo

**Affiliations:** 1 Department of Surgical Oncology, The University of Texas MD Anderson Cancer Center, Houston, TX USA; 2 Platform for Innovative Microbiome and Translational Research (PRIME-TR), The University of Texas MD Anderson Cancer Center, Houston, TX USA; 3 Department of Genomic Medicine, The University of Texas MD Anderson Cancer Center, Houston, TX USA; 4 Department of Translational Molecular Pathology, The University of Texas MD Anderson Cancer Center, Houston, TX USA; 5 Massachusetts General Brigham, Boston, MA USA; 6 Department of Neurosurgery, The University of Texas MD Anderson Cancer Center, Houston, TX USA; 7 Department of Neurosurgery, Baylor College of Medicine, Houston, TX USA; 8 Department of Hematology & Hematopoietic Cell Transplantation, City of Hope, Duarte, CA USA; 9 Department of Cancer Biology, Mayo Clinic, Scottsdale, AZ USA; 10 Advanced Microscopy Core Facility, Department of Epigenetics and Molecular Carcinogenesis, The University of Texas MD Anderson Cancer Center, Houston, TX USA; 11 Electron Microscopy Core Facility,The University of Texas Southwestern, Dallas, TX USA; 12 Department of Medicine, Houston Methodist Research Institute, Houston, TX USA; 13 Department of Medicine, Baylor College of Medicine, Houston, TX USA; 14 Department of Neuro-oncology, The University of Texas MD Anderson Cancer Center, Houston, TX USA; 15 Division of Pathology and Laboratory Medicine, The University of Texas MD Anderson Cancer Center, Houston, TX USA; 16 Cancer Neuroscience Program, The University of Texas MD Anderson Cancer Center, Houston, TX USA; 17 Vivian L. Smith Department of Neurosurgery, McGovern Medical School, The University of Texas Health Science Center at Houston, Houston, TX USA; 18 Department of Genitourinary Medical Oncology, The University of Texas MD Anderson Cancer Center, Houston, TX USA; 19 The University of Texas MD Anderson Cancer Center UTHealth Houston Graduate School of Biomedical Sciences, Houston, TX USA; 20 Brain Tumor Center, The University of Texas MD Anderson Cancer Center, Houston, TX USA; 21 Department of Pediatric Oncology, Dana Farber Cancer Institute, Boston, MA USA; 22 Broad Institute of Massachusetts Institute of Technology and Harvard, Cambridge, MA USA; 23 Department of Medicine, Massachusetts General Hospital, Boston, MA USA; 24 Department of Neurology, Massachusetts General Hospital, Boston, MA USA; 25 Department of Veterinary Medicine and Surgery, Division of Basic Science Research, The University of Texas MD Anderson Cancer Center, Houston, TX USA; 26 Department of Stem Cell Transplantation and Cellular Therapy, The University of Texas MD Anderson Cancer Center, Houston, TX USA; 27 Department of Melanoma Medical Oncology, The University of Texas MD Anderson Cancer Center, Houston, TX USA; 28 Department of Biostatistics, The University of Texas MD Anderson Cancer Center, Houston, TX USA; 29 Department of Immunology, The University of Texas MD Anderson Cancer Center, Houston, TX USA

**Keywords:** CNS cancer, Bacterial host response

## Abstract

Gliomas and brain metastases are associated with poor prognosis, necessitating a deeper understanding of brain tumor biology and the development of effective therapeutic strategies. Although our group and others have demonstrated microbial presence in various tumors, recent controversies regarding cancer-type-specific intratumoral microbiota emphasize the importance of rigorous, orthogonal validation. This prospective, multi-institutional study included a total of 243 samples from 221 patients, comprising 168 glioma and brain metastases samples and 75 non-cancerous or tumor-adjacent tissues. Using stringent fluorescence in situ hybridization, immunohistochemistry and high-resolution spatial imaging, we detected intracellular bacterial 16S rRNA and lipopolysaccharides in both glioma and brain metastases samples, localized to tumor, immune and stromal cells. Custom 16S and metagenomic sequencing workflows identified taxa associated with intratumoral bacterial signals in the tumor microenvironment; however, standard culture methods did not yield readily cultivable microbiota. Spatial analyses revealed significant correlations between bacterial 16S signals and antimicrobial and immunometabolic signatures at regional, neighborhood and cellular levels. Furthermore, intratumoral 16S bacterial signals showed sequence overlap with matched oral and gut microbiota, suggesting a possible connection with distant communities. Together, these findings introduce microbial elements as a component of the brain tumor microenvironment and lay the foundation for future mechanistic and translational studies.

## Abstract (web summary)

A combination of high-resolution spatial imaging, spatial proteomics and transcriptional data reveals sparse and heterogeneous bacterial signals in gliomas and brain metastases.

## Main

Gliomas, including glioblastomas (GBM) and brain metastases (BrM), account for the most common forms of brain malignancies in adults^1,2^. The outcome of patients with primary or metastatic brain tumors remains challenging despite maximal therapy with surgery, radiation and systemic treatments. Understanding the complexities of the brain tumor microenvironment (TME) and identifying factors shaping the tumor immune milieu are critical for advancing our understanding of brain tumor biology and improving the outcome of this disease.

Microbiota has emerged as an important regulator of tumor immunity^3^. Recent studies have demonstrated the presence of microbial cells and genetic material within the TME across various cancer types^4^ and investigated their mechanistic role in cancer progression, specifically in tumor immune infiltration^5–10^. Nevertheless, the presence of a cancer-type-specific microbiota within all major human cancer types has been subject to recent debate^11–13^, largely stemming from the inherent limitations associated with computational methods used to detect and classify low-abundance microbial samples^13^. As a result, findings arising solely from bioinformatic analyses should be interpreted with caution and complemented with validation methods. Importantly, the terms ‘microbiota’ or ‘microbiome’ should be reserved for describing a diverse and active community of microorganisms within an environment, backed by robust evidence.

In this context, initial studies have provided evidence for the presence of microbial elements in brain tumors. Specifically, bacterial nucleic acids have been detected in GBM and central nervous system (CNS) metastases^4,14,15^. Additionally, GBM tumors were found to harbor HLA class II-bound bacterial peptides, recognized by tumor-infiltrating lymphocytes (TILs)^16^. However, whether a diverse microbiota can infiltrate and exist within the brain TME remains an active area of investigation.

In this study, we implemented robust complementary experimental and bioinformatic approaches to investigate the presence of microorganisms and microbial elements in prospective multi-institutional cohorts of individuals with glioma and BrM. We did not observe evidence of a cultivable bacterial community, suggesting the potential absence of a tumor microbiota in brain tumors. However, using extensive high-resolution imaging techniques, we validated the presence of intracellular bacterial elements (RNA, DNA and membrane components) in the brain TME. We further leveraged spatial technologies and demonstrated the correlation of intratumoral bacterial signals with distinct antimicrobial and immunometabolic signatures. Lastly, we used stringent bioinformatic pipelines to characterize the intratumoral bacterial signals and demonstrate a correlation with distant microbiota.

## Results

### Bacterial 16S signals are detectable in primary and metastatic brain tumors

**Fig. 1:**
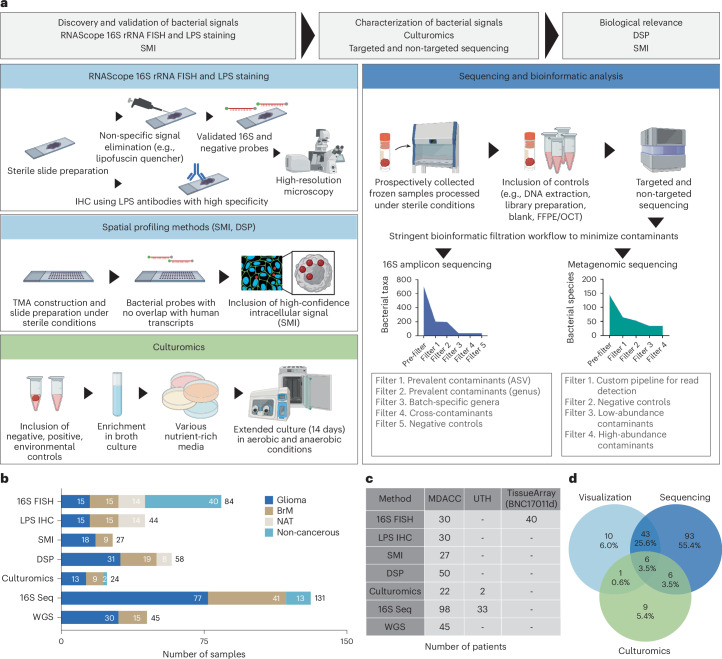
Study workflow. **a**, Schematic of the study workflow. To assess the presence and correlation of bacteria and bacterial elements in brain tumors, we combined integrative methods, including RNAScope FISH and SMI for detection and validation of bacterial signals, culturomics and bioinformatic analyses for taxonomic characterization of intratumoral bacterial signals and spatial technologies to evaluate biological relevance within the TME. Stringent workflows were developed to enhance each methodology. Schematics were created using BioRender.com. **b**,**c**, Samples from 221 patients were included in this study, with distribution shown by analytical method (**b**) and institution (**c**). **d**, When sufficient tissue was available, samples were analyzed using at least two methods in the following categories: visualization (FISH, SMI and DSP); sequencing (16S rRNA amplicon and metagenomic shotgun sequencing); and culturomics. The Venn diagram depicts the number of samples analyzed across each methodological category. Seq, sequencing; WGS, whole-genome sequencing.

To determine and validate the presence, distribution and biological relevance of bacteria and bacterial elements in primary and metastatic brain tumors, we applied a stringent workflow integrating complementary experimental and bioinformatic approaches, including fluorescence in situ hybridization (FISH), immunohistochemistry (IHC) and spatial molecular imaging (SMI) for signal detection; culturomics and bioinformatic analyses for characterization; and SMI and digital spatial profiling (DSP) for functional analysis (Fig. 1a). The study included 243 tissue samples from a total of 221 patients (168 tumor: 113 glioma, 55 BrM; 22 normal tissue adjacent to tumor (NAT); and 53 non-cancerous brain tissue) (Fig. 1b,c). When sufficient material was available, tumor samples were analyzed using at least two methods in the following categories: visualization, sequencing and culturomics (Fig. 1d and Supplementary Table 1). In total, 33 of 113 glioma and 23 of 55 BrM samples were assessed by methods in at least two different categories.

**Fig. 2:**
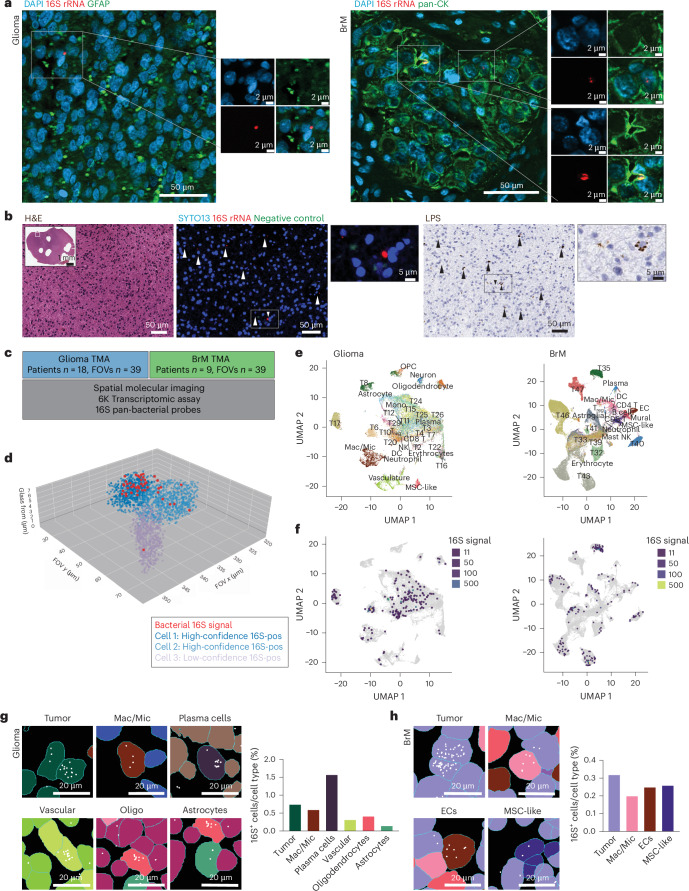
Bacterial signals are detectable in primary and metastatic brain tumors. **a**, RNAScope 16S rRNA FISH of glioma (left) and BrM (right). Representative images of 15 glioma and 15 BrM samples (female 40%, age range 20–71 years; one full section slide per patient). Nuclei, DAPI, blue; bacterial 16S rRNA, red; GFAP, pan-cytokeratin (pan-CK), green in glioma and BrM, respectively. **b**, Representative images of H&E staining, 16S rRNA FISH and LPS IHC staining of a brain tumor sample (patient cohort same as **a**; one sequential full section slide per patient). Nuclei, SYTO13, blue; bacterial 16S rRNA, red; negative control, green. White and black arrows mark 16S rRNA and LPS signals with proximity to nuclei, respectively. **c**, Schematic demonstrating the experimental workflow for SMI (CosMx platform). Patients: glioma *n* = 19, BrM *n* = 9, female 37.1%, age range 28–80 years; FOVs *n* = 39 per TMA. **d**, Three-dimensional reconstruction of cells containing high-confidence intracellular 16S signal (light and dark blue) and low-confidence low-level 16S signal (purple). Bacterial 16S signals, red; human transcripts identified in each cell, purple and blue. **e**, UMAP plot of single-cell clustering of glioma and BrM samples. Each cell cluster is represented by a different color and labeled. **f**, UMAP plot of high-confidence 16S signal distribution in each cluster and corresponding normalized counts. **g**,**h**, Representative images and bar plots demonstrating the percentage of high-confidence 16S-positive cells within glioma (**g**) and BrM (**h**) TME. 16S signal, white. Images were obtained using napari software. DC, dendritic cell; ECs, endothelial cells; Mac/Mic, macrophage/microglia; MSC, mesenchymal stem cell; NK, natural killer cell; Oligo, oligodendrocytes; OPC, oligodendrocyte progenitor cell; T_reg_, regulatory T cell.

**Extended Data Fig. 1:**
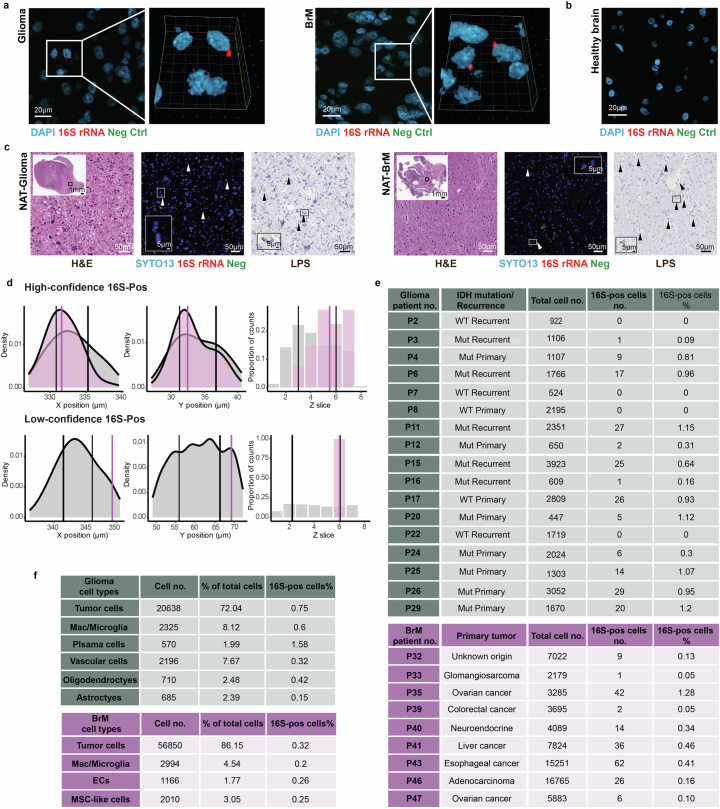
Distribution of 16S signal within cells and across patient samples. **a,b**, RNAScope 16S rRNA FISH of (**a**) glioma, BrM, and (**b**) healthy brain tissue; bacterial 16S rRNA, red; negative control probe, green; DAPI, blue, 20X objective; 3D reconstructions, 63X objective (patients: glioma n = 15, BrM n = 15; female 40%, age range 20-71 years; 1 full section slide per patient; Normal n = 40; female 32.9%, age range 15-54 years; 1 TMA, 2 cores per patient). **c**, RNAScope 16S rRNA FISH and Lipopolysaccharide (LPS) staining of normal tissue adjacent to tumor (NAT) in glioma and BrM samples (patients: glioma n = 13, BrM =3, female 57%, age range 28-71 years; 1 full section slide per patient). Arrows demonstrate staining with intracellular pattern. Nuclei, SYTO13, blue; 16S rRNA, red; negative control, green. **d**, Density plots demonstrating the distribution of the bacterial 16S median (red line) in relationship to all human transcripts within a cell at X, Y, Z axes. The two black lines indicate where 50% of human transcripts are located. high-confidence cell, upper panels; low-confidence cell, lower panels. **e**, Tables demonstrating the total cell number per patient, and total number and percentage of 16S-positive cells per patient. **f**, Table demonstrating the total number of cells per cell type, percentage of each cell type among the total cells, and the percentage of 16S-pos cells for each cell type.

We first conducted RNAScope FISH on whole-tissue sections from 15 glioma and 15 BrM samples, using a validated 16S rRNA pan-bacterial probes^7^ alongside a negative control probe. Distinct 16S rRNA signals were detected in 11 glioma and nine BrM tumors (Supplementary Table 1). *z*-stack imaging detected 16S rRNA signals in close proximity to the nucleus, suggestive of intracellular localization (Extended Data Fig. 1a). Co-staining with glial fibrillary acidic protein (GFAP; glioma) or pan-cytokeratin (BrM) membrane markers revealed diverse localization patterns of bacterial 16S rRNA, including cytoplasmic, membrane-adjacent and extracellular distributions (Fig. 2a). Notably, 16S rRNA signals varied in size and morphology, with some consistent with intact bacteria (approximately 2 μm) and others exhibiting punctate patterns (Fig. 2a), suggesting the potential presence of both intact bacterial cells and fragmented bacterial 16S rRNA. To further investigate the presence of bacterial elements, we performed lipopolysaccharide (LPS) staining on consecutive tissue sections to those used for 16S rRNA FISH. LPS was detected in 13 glioma and nine BrM samples, with staining patterns suggesting both intracellular and extracellular localization (Fig. 2b and Supplementary Table 1). Notably, 16S rRNA FISH and LPS staining results were concordant in 22 of 30 samples (positive *n* = 18 and negative *n* = 4 by both methods; Supplementary Table 1). Discordant findings in the remaining samples may reflect differences in bacterial RNA and protein stability or degradation across sequential tissue sections.

To evaluate whether these bacterial signals were specific to tumor tissue, we next examined non-tumor controls. Bacterial 16S rRNA signal was not detected in a fresh-cut healthy brain tissue microarray (TMA; TissueArray, BNC17011d; Extended Data Fig. 1b). We also assessed 16S rRNA signal and LPS staining in NAT (*n* = 14). Although 16S rRNA signal was detected in only four samples, LPS staining was positive in 12 of 14 samples and was observed in both perivascular and parenchymal regions (Extended Data Fig. 1c).

To assess the identified bacterial 16S signals at higher resolution and to examine their spatial distribution within the brain TME, we conducted SMI using the CosMx platform (Bruker Spatial Biology). SMI was performed on two TMAs constructed from glioma and BrM tumors (Supplementary Table 2) and used the Human 6K Discovery Panel along with a customized panel of pan-bacterial 16S probes (Fig. 2c and Methods).

Intratumoral 16S signal was detected both intracellularly and within the extracellular environment. Although the potential biological significance of extracellular 16S signals cannot be disregarded, current technologies are limited in accurately distinguishing between true extracellular 16S rRNA signal and potential contamination introduced during tissue processing (for example, tissue fixation and embedding). Therefore, we exclusively considered intracellular 16S signals for analysis. To ensure true intracellular localization, we excluded cells where bacterial 16S signals were near the cell membrane, as such proximity combined with the inherent limitations associated with cell segmentation could create false-positive intracellular patterns. Thus, only cells in which the median bacterial 16S position was within the central 50% of all transcript positions across all *x*, *y* and *z* axes (that is, centered within the cell body) were considered high-confidence 16S-positive cells (Fig. 2d, Extended Data Fig. 1d and Supplementary Video 1). Of the 28,177 and 65,993 cells analyzed across all cores in glioma and BrM TMAs, we identified a total of 182 (0.65%) and 198 (0.3%) cells with high-confidence intracellular 16S signal, respectively.

The intracellular 16S signal exhibited heterogeneous distribution patterns across and within the analyzed tumors and fields of view (FOVs), suggesting a non-random distribution. First, the different tumor samples exhibited variation in the intracellular 16S signal count per patient (glioma, average: 1,098.2, range 25–4,236; BrM, average: 943.6, range 30–2,706) and the total number of 16S-positive cells detected per patient (glioma, range 0–27; BrM, range 1–62) (Extended Data Fig. 1e). This distribution pattern was not related to tissue core position on the TMA (Supplementary Table 2). Furthermore, intracellular 16S signal in glioma and BrM exhibited 16-fold and 67.5-fold higher density, respectively, compared to 16S signal in the extracellular environment, which is likely the result of contamination with expected random tissue distribution (median intracellular signal density per μm^2^: glioma 7.52 × 10^−3^, BrM 7.56 × 10^−3^; median extracellular 16S signal per μm^2^: glioma 4.78 × 10^−4^, BrM 1.13 × 10^−4^).

**Extended Data Fig. 2:**
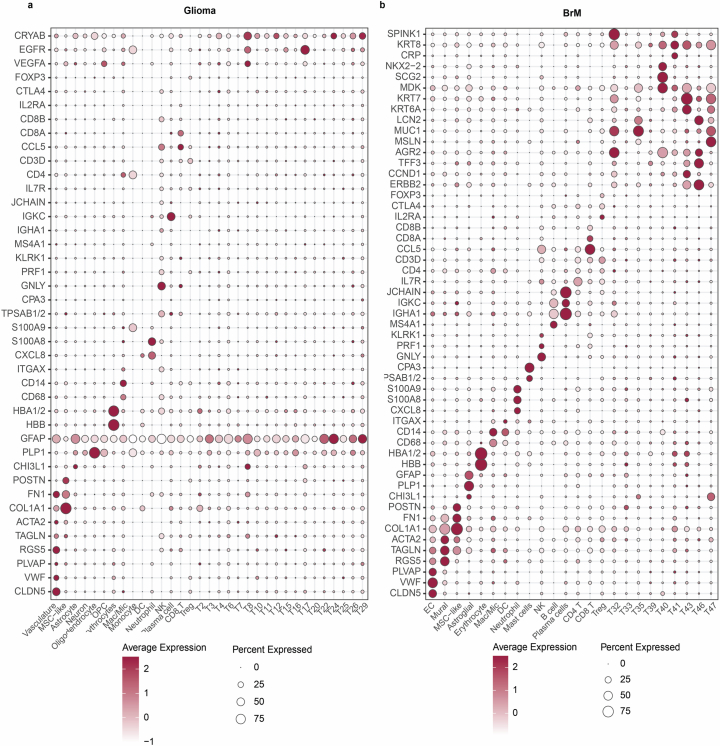
Expression of transcripts across cell clusters. **a,b**, Bubble plots demonstrating the marker transcripts in each cell cluster identified for (**a**) glioma and (**b**) BrM. MSC, mesenchymal stem cell; Mac/Mic, macrophage/microglia; DC, dendritic cell; NK, natural killer cell; Oligo, oligodendrocyte; OPC, oligodendrocyte progenitor cell; ECs, endothelial cells; Treg, regulatory T cells; T, tumor.

Our observations prompted us to examine the cell types harboring intracellular 16S signal. In both glioma and BrM TMAs, intracellular bacterial 16S signals were detected in tumor cells as well as in various immune and stromal cells (Fig. 2e–h and Extended Data Figs. 1f and 2). Together, our comprehensive molecular analysis of glioma and BrM samples using 16S RNAScope FISH, LPS staining and high-resolution SMI demonstrated that intracellular bacterial elements can be detected in the brain TME.

Next, we assessed whether the bacterial signal detected within the brain TME could be attributed to cultivable bacteria. We prepared homogenates from nine freshly resected tumors, 13 fresh-frozen tumor samples and two non-cancerous fresh-frozen brain samples from individuals with epilepsy (Supplementary Table 1; female 50%, age range 26–77 years). All fresh-frozen samples were assessed using SMI, 16S amplicon sequencing and/or metagenomic shotgun sequencing and were confirmed to contain bacterial 16S signal (Supplementary Table 3). In freshly resected tumors, sufficient tissue was available in three samples to conduct 16S rRNA digital polymerase chain reaction PCR (dPCR) post-culture, which confirmed the presence of bacterial 16S copies (2.5–16 copies per microliter). Homogenates were plated on nutrient-rich media, with or without pre-enrichment in nutrient-rich broth, and subjected to both anaerobic and aerobic conditions. Across all conditions, no bacterial growth was observed after 14 days of culture. These findings suggest that brain tumor and non-cancerous brain samples may lack readily cultivable bacteria, as assessed by standard culture techniques.

### Distinct bacterial signatures were identified in brain tumors compared to non-cancerous brain tissue

**Fig. 3:**
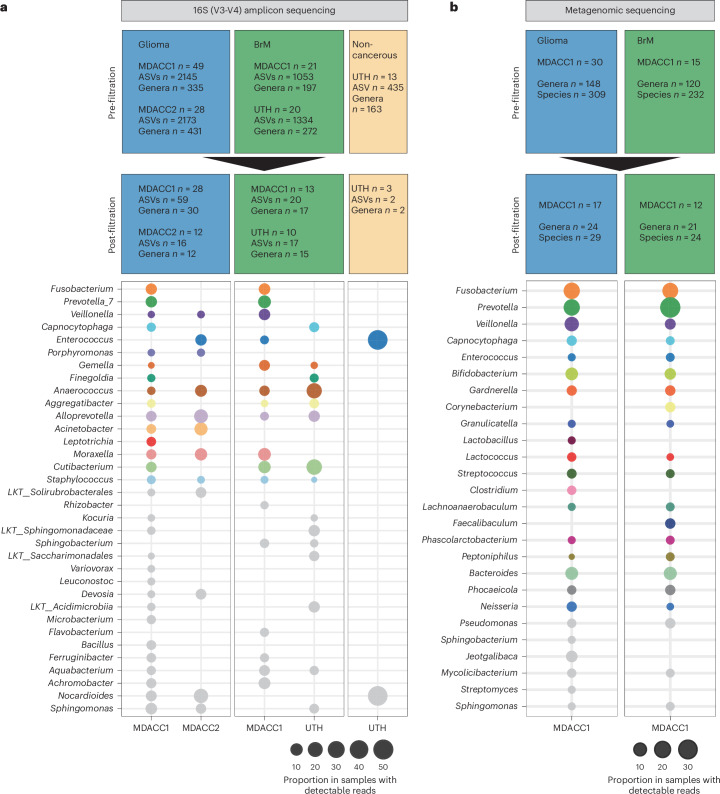
Distinct bacterial signatures were identified in brain tumors compared to non-cancerous brain tissue. **a**, Tumor samples (glioma *n* = 77, BrM *n* = 41, fresh-frozen *n* = 109, formalin-fixed paraffin-embedded (FFPE), *n* = 5, OCT (Optimal cutting temperature), *n* = 4; female 39.8%, age range 20–83 years) and non-cancerous brain tissue (fresh-frozen, *n* = 13; female 23.1%, age range 20–73 years) were assessed using 16S (V3-V4) rRNA amplicon sequencing. Bubble plot demonstrates the proportion of bacterial taxa identified among samples with detectable bacterial signal in glioma, BrM and non-cancerous prospective cohorts. Taxa identified as potential environmental contaminants after filtration are presented in gray. Tables present the number of samples, ASVs and genera identified pre-filtering and post-filtering. **b**, Glioma (*n* = 30) and BrM (*n* = 15) samples were assessed using metagenomic shotgun sequencing (fresh-frozen *n* = 45; female 44.4%, age range 26–77 years). Bubble plot demonstrates the proportion of bacterial taxa identified among samples with detectable bacterial signal, presented at genus level. Taxa identified as potential environmental contaminants after filtration are presented in gray. Tables present the number of samples, genera and species identified pre-filtering and post-filtering.

To further characterize the intratumoral bacterial signals, we assembled prospective cohorts of glioma and BrM tumor samples from patients at MD Anderson Cancer Center (MDACC1 and MDACC2 cohorts) and the University of Texas Health Science Center at Houston (UTH cohort) (Fig. 3a and Supplementary Table 3), which were analyzed using 16S (V3-V4) rRNA amplicon sequencing. For comparison, we included a cohort of non-cancerous brain tissue resected as part of a procedure for drug-resistant epilepsy (UTH non-cancerous brain). Two glioma samples from the MDACC1 cohort failed to yield sequencing reads and were excluded from analysis.

**Extended Data Fig. 3:**
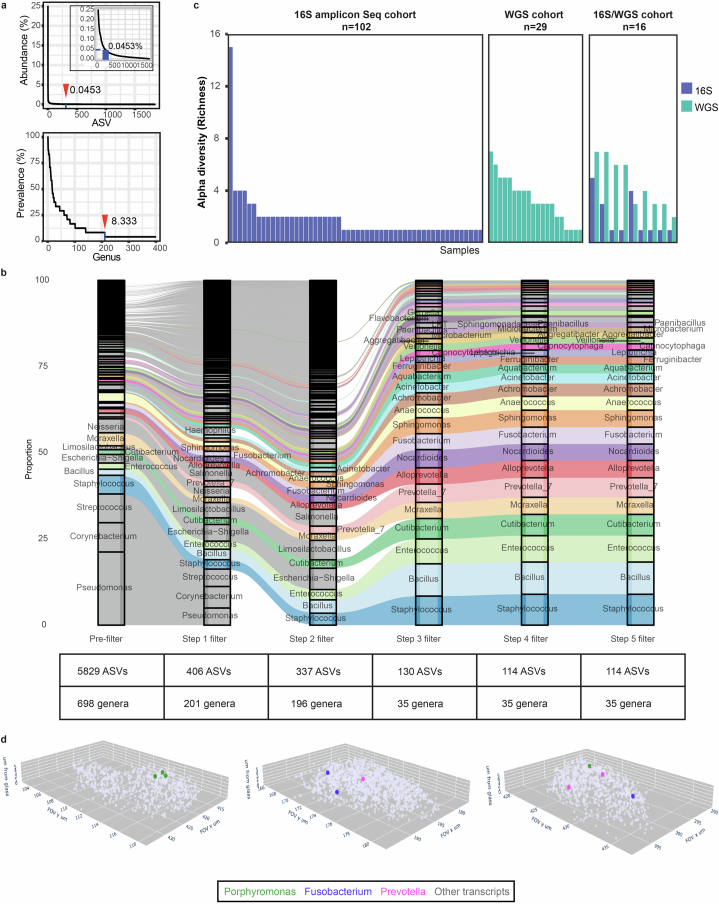
Filtration of 16S rRNA and metagenomic shotgun sequencing datasets. **a**, Proportion of ASVs and prevalence of genera identified in controls pooled from all sequencing batches (controls n = 24). Inflection point was calculated via ChangePoint analysis. The inner proportion plot depicts a higher magnification of the inflection point. Red arrow, inflection point. **b**, Alluvial plot demonstrating the taxa identified across all glioma, BrM, and non-cancerous brain samples pre-filtration and after each filtration step. Table demonstrates the number of ASVs and genera present at each filtration step. **c**, Histograms demonstrate bacterial richness (alpha diversity) in brain tumors post-filtration, based on 16S amplicon sequencing or metagenomic shotgun sequencing. Both methods were applied to 16 samples, with 9 samples yielding reads by both methods and presented separately. **d**, 3D reconstruction of cells with intra-cellular genus-specific bacterial signal. *Porphyromonas*, green; *Fusobacterium*, blue; *Prevotella*, magenta; human transcripts, gray.

Bioinformatic analysis of 16S datasets from samples anticipated to have no or low-abundance bacterial signal, such as brain tissue, poses challenges, largely owing to contamination effects^13^. To detect and minimize the impact of potential contaminants in our analysis, we applied a series of five filtration steps that aimed to remove the most common types of contamination (Methods). After this filtration process, 46.6% of the tumor samples (55 out of 118) and 76.9% of the non-cancerous brain samples (10 out of 13) did not retain any detectable bacterial signal (Fig. 3a). Across the remaining samples, a total of 34 bacterial taxa were identified (observed genera per sample 1–15; Fig. 3a, Extended Data Fig. 3a,b and Supplementary Table 3). Notably, consistent with our 16S FISH, minimal signal was detected in non-cancerous brain tissue. These findings collectively suggest the tumor specificity of the identified intratumoral 16S signal. Among the identified bacterial taxa, 16 taxa were associated with human commensal microbiota, representing potential biologically meaningful signal (amplicon sequence variant (ASV): tumor *n* = 59, non-cancerous brain *n* = 2, Gram-negative taxa *n* = 12, Gram-positive taxa *n* = 4); however, 18 taxa were found to be known environmental contaminants (ASV *n* = 53; Fig. 3a). These results highlight the inherent limitations associated with bioinformatic analysis of low-abundance samples, indicating that even stringent filtration methods cannot eliminate all contaminants. Consequently, taxonomic data obtained from bioinformatic analyses of samples with low biomass warrant validation.

We further asked if the taxa identified by 16S rRNA gene sequencing could be validated with broader gene coverage. We conducted metagenomic shotgun sequencing on 45 tumor samples (glioma *n* = 30, BrM *n* = 15). Non-human reads comprised an average of 0.056% of the total reads (range 0.03–0.09%), of which an average of 0.46% was found to be bacterial (range 0.002–2.13%). To complement the 16S (V3-V4) coverage obtained by 16S rRNA sequencing, metagenomic sequencing was filtered against the SILVA ribosomal RNA gene database^17^, ensuring that the detected taxonomies were derived from non-ribosomal bacterial genes. We applied a filtration workflow to minimize the inclusion of reads likely arising from contamination (Methods), which eliminated 43% and 20% of glioma and BrM samples, leaving a total of 29 and 24 species (24 and 21 genera) in 17 glioma and 12 BrM samples, respectively (observed species per sample: 1–7; Fig. 3b and Supplementary Table 4). This analysis obtained non-ribosomal coverage for five of the 16 bacterial genera identified using 16S rRNA gene sequencing, including *Fusobacterium*, *Veillonella*, *Prevotella*, *Capnocytophaga* and *Enterococcus* (Fig. 3b). Additionally, metagenomic analysis was concordant with our 16S amplicon sequencing in detecting the presence of bacterial signal in nine of the 16 samples assessed by both methods (Supplementary Table 1 and Extended Data Fig. 3c), with a moderate correlation in bacterial richness (Spearman’s *ρ* = 0.59, *P* = 0.09). Seven samples had detectable reads by metagenomic sequencing but not by 16S amplicon sequencing. Together, these findings provide broader gene coverage of bacterial taxa in brain tumors, although complete bacterial genomes could not be recovered, likely owing to the low biomass.

To complement our bioinformatic approach, we used SMI to validate the intracellular localization of the bacterial taxa identified through targeted sequencing, using three sets of genus-specific probes for *Fusobacterium*, *Porphyromonas* and *Prevotella*. We detected intracellular signals from all three genera (Extended Data Fig. 3d), albeit at a lower frequency compared to pan-bacterial 16S signal (Fig. 2d).

Collectively, these findings reveal distinct bacterial taxa in gliomas and BrMs and confirm that at least a subset exhibits intracellular localization. Nevertheless, determining the significance and generalizability of individual taxa will require larger-scale studies.

### Tumor regions with high 16S signal are enriched in antimicrobial signatures

**Fig. 4:**
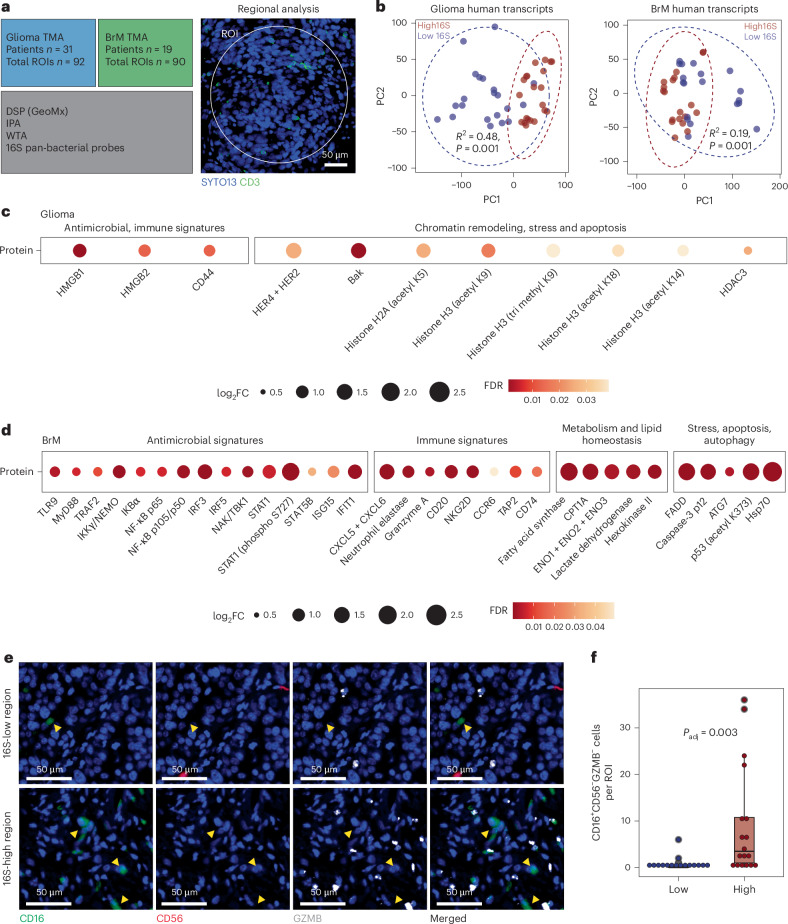
16S-high tumor regions are enriched in antimicrobial signatures. **a**, Schematic demonstrating the experimental workflow for DSP (GeoMx platform). Patients: glioma *n* = 31, BrM *n* = 19; female 34%, age range 20–81 years. Representative image of an ROI containing CD3^+^ cells, taken using the GeoMx platform. **b**, PCA demonstrating the clustering of human transcripts identified in glioma (*R*^2^ = 0.48, *P* = 0.001, two-sided PERMANOVA) and BrM (*R*^2^ = 0.19, *P* = 0.001, two-sided PERMANOVA) TMAs based on categorical 16S status analyzed by DSP, background subtracted and Q3 normalized. Low-16S ROIs, blue; high-16S ROIs, red. **c**, Differentially expressed proteins in glioma 16S-high tumor regions (two-sided LMM, Benjamini–Hochberg test for multiple comparison adjustment, adjusted *P* < 0.05, log_2_FC > 0.58). **d**, Differentially expressed proteins in BrM 16S-high tumor regions (two-sided LMM, Benjamini–Hochberg test for multiple comparison adjustment, adjusted *P* < 0.05, log_2_FC > 0.58). **e**, Multiplex fluorescence IHC of BrM samples. Representative images of 16S-low (top panels) and 16S-high (bottom panels) are demonstrated. CD16, green; CD56, red, GZMB, white; yellow arrows, CD16^+^ cells, ×40 magnification. **f**, Bar plot demonstrating the differential abundance of CD16^+^CD56^−^GZMB^−^ populations in 16S-low (patients *n* = 6, ROIs *n* = 16) and 16S-high (patients *n* = 5, ROIs *n* = 18) regions in BrM TMA (two-sided LMM of log_2_-transformed counts, Benjamini–Hochberg test for multiple comparison adjustment, adjusted *P* = 0.012, untransformed counts presented). Box plots show median (center line), interquartile range (IQR) (box bounds, 25th and 75th percentiles) and smallest and largest values within 1.5× the IQR (whiskers). Outliers are plotted beyond the whiskers. FDR, false discovery rate; *P*_adj_, adjusted *P* value; PC, principal component.

**Extended Data Fig. 4:**
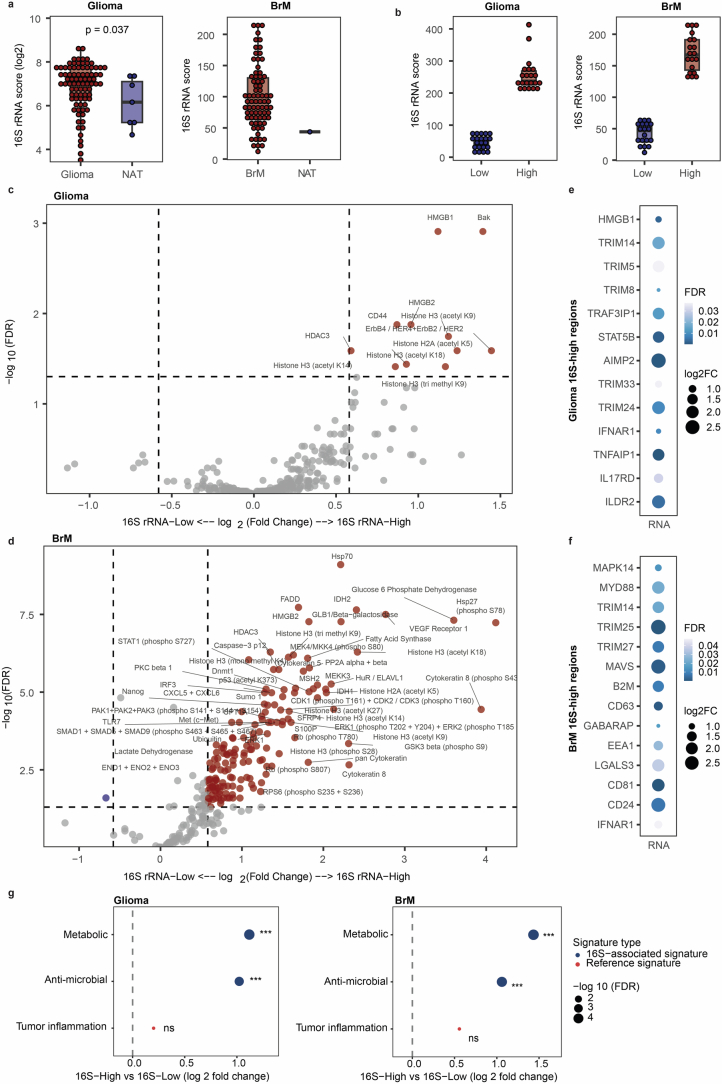
Distribution of 16S signal and the associated enriched proteins and transcripts. **a**, Bacterial score in glioma and BrM tumor regions compared to the normal tissue adjacent to tumor (glioma NAT n = 7, p value = 0.037, BrM NAT n = 1). Two-sided Linear Mixed Model (LMM), Benjamini-Hochberg test for multiple comparison adjustment, adjusted P value < 0.05. Boxplots show median (center line), interquartile range (box bounds, 25^th^ and 75^th^ percentiles), and smallest and largest values within 1.5× the IQR (whiskers). Outliers are plotted beyond the whiskers. **b**, Bacterial score in glioma and BrM 16S-high (1^st^ quartile; glioma patients n = 11, ROI n = 21; BrM patients n = 11, ROI n = 19) vs. 16S-low tumor regions (4^th^ quartile; glioma patients n = 12, ROI n = 21; BrM patients n = 6, ROI n = 19). Boxplots show median (center line), interquartile range (box bounds, 25^th^ and 75^th^ percentiles), and smallest and largest values within 1.5× the IQR (whiskers). Outliers are plotted beyond the whiskers. **c,d**, Volcano plot demonstrating the proteins enriched in (**c**) glioma and (**d**) BrM 16S-high tumor regions. Two-sided LMM, Benjamini-Hochberg test for multiple comparison adjustment, adjusted P value < 0.05, log2FC > 0.58. **e,f**, Differentially expressed transcripts in (**e**) glioma and (**f**) BrM 16S-high tumor regions. Two-sided LMM, Benjamini-Hochberg test for multiple comparison adjustment, adjusted P value < 0.05, log2FC > 1.5. **g**, Correlation of the identified anti-microbial, metabolic signatures, and reference tumor inflammation signatures with the 16S bacterial signal.

We next used DSP (GeoMx platform; Bruker Spatial Biology) to examine the association of intratumoral 16S signal with protein and transcript signatures in the brain TME. DSP was conducted on the previously described glioma and BrM TMAs using the GeoMx Immuno-Oncology Proteome Atlas (IPA), the Whole Transcriptomic Assay (WTA) (Bruker Spatial Biology) and custom pan-bacterial 16S probes (Fig. 4a). Notably, bacterial 16S signal was significantly lower in normal brain tissue adjacent to tumor compared to tumor tissue (Extended Data Fig. 4a).

To investigate the spatial relationship of bacterial rRNA with tumor protein and transcript signatures, we calculated the geometric mean of bacterial signal obtained from 16S probes and annotated regions of interest (ROIs) as 16S-low (first quartile) or 16S-high (fourth quartile) (Extended Data Fig. 4b). Unsupervised principal component analysis (PCA) of human transcripts suggested distinct clustering of 16S-high tumor ROIs compared to 16S-low tumor ROIs (Fig. 4b). To validate this, we used a linear mixed model (LMM) (Methods) to identify differentially enriched transcripts and proteins in 16S-high regions compared to 16S-low regions (Supplementary Table 5).

In both glioma and BrM 16S-high tumor regions, we identified proteins and transcripts linked to antimicrobial response (Fig. 4c,d, Extended Data Fig. 4c–f and Supplementary Table 5). In glioma, we observed a protein enrichment of damage-associated molecular pattern (DAMP) molecules, including HMGB1 and HMGB2, which interact with pattern recognition receptors (PRRs) such as Toll-like receptors (TLRs)^18^ (Fig. 4c). In BrM, 16S-high regions exhibited a protein enrichment of TLR9 (Fig. 4d), a key PRR for the detection of intracellular microbial nucleic acids^19,20^, as well as the TLR9 downstream canonical pathway, supported by the upregulation of MyD88, members of the NF-κB family commonly activated by TLRs (RelA (also known as p65) and NF-κB2 (also known as p105/p50)) and interferon regulatory factors (IRFs)^21^, among others (Fig. 4d). Differentially abundant transcripts in 16S-high regions were consistent with these protein signatures, including transcripts related to TLR and NF-κB pathways and other transcripts linked to response to intracellular microbial elements, such as TRIM family^22^ (Extended Data Fig. 4e,f).

In BrM 16S-high tumor regions, we also observed an upregulation of neutrophil chemoattractants, neutrophil enzymes and antigen presentation proteins (Fig. 4d). To further explore the cell composition within the 16S-high regions, we conducted multiplex immunofluorescence staining on consecutive TMA sections using myeloid and lymphoid panels (Methods) and found a significant enrichment of CD16^+^CD56^−^GZMB^−^ cells (adjusted *P* = 0.003, log_2_ fold change (log_2_FC) = 2.38; Fig. 4e,f), potentially representing neutrophils.

In addition to antimicrobial signatures, we also observed an enrichment of chromatin remodeling proteins in glioma (Fig. 4c) as well as lipid metabolism and cell stress response proteins and transcripts in both glioma and BrM 16S-high tumor regions (Fig. 4d and Extended Data Fig. 4e,f).

Notably, the antimicrobial, immune and metabolic signatures associated with the intratumoral 16S signal were not linked to a generalized inflammatory environment, as they showed no significant correlation with other tumor inflammation signatures, including IL-6 and STAT3 pathways, macrophage activity and chronic inflammation (Extended Data Fig. 4g). Collectively, these findings provide evidence of antimicrobial, immune and metabolic signatures associated with intratumoral 16S-high regions in both BrM and glioma tumors.

### 16S-positive tumor cells and neighborhoods exhibit distinct transcriptional profiles

Next, we leveraged SMI to validate the regional analyses from DSP at higher resolution, at the single-cell and neighborhood levels. Participants with more than 20 16S-positive tumor cells were included for analysis (glioma, *n* = 3; BrM, *n* = 4).

**Fig. 5:**
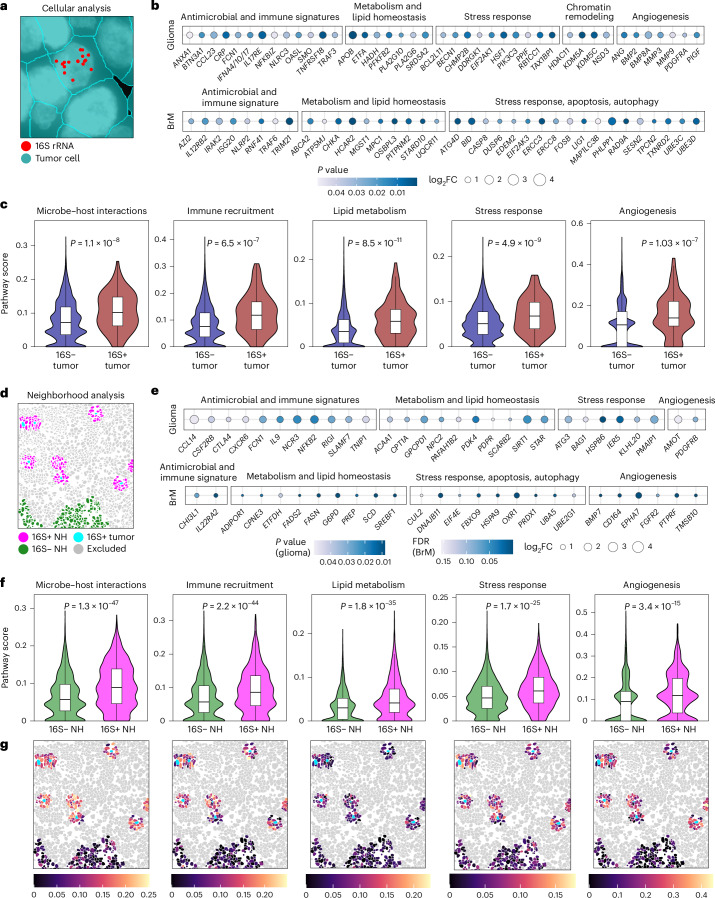
16S-positive tumor cells and neighborhoods exhibit distinct transcriptional profiles. **a**, Representative image of 16S-positive tumor cells assessed for differential transcriptional profile compared to 16S-negative cells; patients with more than 20 16S-positive tumor cells were included (patients with glioma *n* = 3 (P11, P15 and P26), 16S-positive tumor cells *n* = 68, 16S-negative tumor cells *n* = 6,559; patients with BrM *n* = 4 (P35, P41, P43 and P46), 16S-positive tumor cells *n* = 155, 16S-negative tumor cells *n* = 35,221). Tumor cells, teal; 16S signal, red. Image was obtained using napari software. **b**, Bubble plots demonstrating enriched transcripts in 16S-positive tumor cells across patients with glioma and BrM (two-sided LMM, Benjamini–Hochberg test for multiple comparison adjustment, *P* < 0.05, log_2_FC > 0.58). **c**, Violin plots demonstrating pathway scores in 16S-positive and 16S-negative tumor cells in patients with BrM (two-sided LMM, Benjamini–Hochberg test for multiple comparison adjustment, *P* < 0.05, log_2_FC > 0.58). Violin plots show smoothed AUCell score distributions, overlaid with box plots indicating the median (center line), interquartile range (IQR) (box bounds, 25th and 75th percentiles) and smallest and largest values within 1.5× IQR (whiskers). **d**, Representative image of neighborhoods (NHs) within 30-µm radius surrounding 16S-positive tumor cells and those surrounding 16S-negative tumor cells (patients with glioma *n* = 3 (P11, P15 and P26), cells within 16S-positive neighborhoods *n* = 731, cells within 16S-negative neighborhoods *n* = 492; patients with BrM *n* = 3 (P41, P43 and P46), cells within 16S-positive neighborhoods *n* = 2,093, cells within 16S-negative neighborhoods *n* = 8,134). Teal, 16S-positive tumor cell; magenta, 16S-positive neighborhoods; green, 16S-negative neighborhoods. Cells more than 30 μm away from a tumor cell and those between 30 μm and 100 μm of the closest 16S-positive cell were excluded (gray). **e**, Bubble plots demonstrating transcripts enriched in combined 16S-positive neighborhoods from patients with glioma and BrM (two-sided LMM, Benjamini–Hochberg test for multiple comparison adjustment, *P* < 0.05, log_2_FC > 0.58). **f**, Violin plots demonstrating pathway scores in 16S-positive compared to 16S-negative neighborhoods in patients with BrM (two-sided LMM, Benjamini–Hochberg test for multiple comparison adjustment, *P* < 0.05, log_2_FC > 0.58). Violin plots show smoothed AUCell score distributions, overlaid with box plots indicating the median (center line), IQR (box bounds) and values within 1.5× IQR (whiskers). **g**, An example of spatial distribution of different pathways in 16S-positive and 16S-negative neighborhoods, demonstrated for BrM P43.

We first conducted differential gene expression analysis across samples from individuals with glioma and BrM to identify shared tumor-intrinsic transcriptional patterns associated with 16S signal in each tumor type (Fig. 5a). Consistent with DSP analyses, we identified transcripts related to antimicrobial and immune responses, including PRRs, NF-κB and TRIM family members and interleukin signaling as well as transcripts involved in lipid metabolism, cellular stress and apoptosis/autophagy in both glioma and BrM (Fig. 5b and Supplementary Table 6). In glioma, 16S-positive cells also showed upregulation of angiogenesis-related transcripts, potentially linked to the antimicrobial and innate immune signatures^23,24^, along with transcripts associated with chromatin remodeling, axonogenesis and neuroinflammation (Supplementary Table 6).

**Extended Data Fig. 5:**
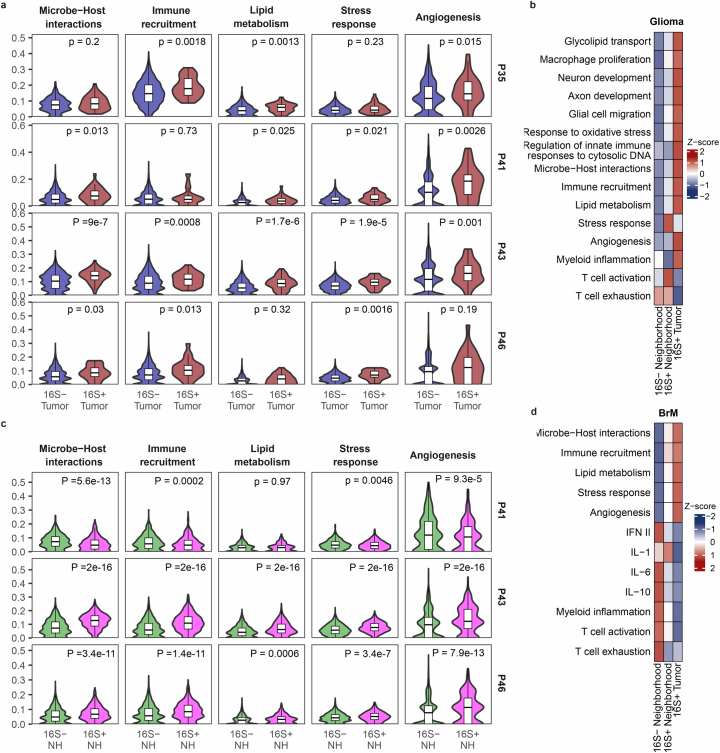
16S-pos tumor cells and neighborhoods exhibit distinct transcriptional scores, per patient. **a**, Violin plots demonstrating biological pathway scores in 16S-positive and -negative tumor cells in individual patients (BrM 16S-pos/16S-neg tumor cells: P35 40/2247, P41 35/5776, P43 58/12609, P46 22/14589). Two-sided Wilcoxon signed-rank test. Violin plots show smoothed AUCell score distributions, overlaid with boxplots indicating the median (center line), interquartile range (box bounds, 25th and 75th percentiles), and smallest and largest values within 1.5× IQR (whiskers). **b**, heatmap demonstrating the z-score of 16S-associated pathways in glioma 16S-positive tumor cell and neighborhoods and 16S-negative neighborhoods. **c**, violin plots demonstrating biological pathway scores in 16S-positive and -negative neighborhoods in individual patients (BrM cell number in 16S-pos/16S-neg neighborhoods: P41 557/1061, P43 1024/1800, P46 512/5273). Two-sided Wilcoxon signed-rank test. Violin plots show smoothed AUCell score distributions, overlaid with boxplots indicating the median (center line), interquartile range (box bounds, 25th and 75th percentiles), and smallest and largest values within 1.5× IQR (whiskers). **d**, heatmap demonstrating the z-score of 16S-associated and general inflammatory control pathways in 16S-positive tumor cell and neighborhoods and 16S-negative neighborhoods (containing 16S-negative tumor cells).

To identify broader biological patterns, we conducted pathway analysis using curated gene sets derived from Gene Ontology Biological Process (GOBP) and Reactome annotations (Methods). These custom modules reflected key processes highlighted by DSP, including microorganism–host interactions, immune recruitment, lipid metabolism, cellular stress responses and angiogenesis. In BrM, pathway scores were significantly higher in the combined 16S-positive tumor cells from all four patients, indicating that the associated pathways reflect a coordinated transcriptional program rather than isolated gene-level changes (Fig. 5c). At the individual patient level, pathway scores were significantly different in at least three of four patients (Extended Data Fig. 5a), suggesting limited interpatient variability in the observed transcriptional patterns. Together, these findings demonstrate a layer of tumor cell heterogeneity in the BrM TME that is microbial context dependent; however, the causal role of microbial elements in this setting remains to be explored. Assessment of these predefined pathways in glioma tumor cells did not yield consistent differences owing to high interpatient variability; however, other gene sets related to glial and neuronal processes were also found to be upregulated, suggesting tumor- type-specific associations with 16S signals (Extended Data Fig. 5b).

We next analyzed shared transcriptional profiles of glioma and BrM neighborhoods within a 30-µm radius of 16S-positive tumor cells (2–3 surrounding cell rows) compared to 16S-negative neighborhoods, including only samples with sufficient eligible regions (Methods and Fig. 5d). Consistent with our regional analyses, combined neighborhoods surrounding 16S-positive cells in glioma and BrM samples exhibited an upregulation of transcripts involved in cytokine signaling and immune recruitment as well as transcripts related to lipid metabolism, cellular stress and angiogenesis (Fig. 5e and Supplementary Table 6). Pathway analysis in BrM demonstrated higher scores across all predefined pathways in both the combined dataset and individual patients, except lipid metabolism in patient 41 (P41) (Fig. 5f,g and Extended Data Fig. 5c). To assess whether the pathways identified in BrM reflect a general inflammatory state, we examined associations with type II interferon (IFN-II), IL-1, IL-6 and IL-10 signaling, myeloid inflammation and T cell activity. None was significantly enriched in 16S-positive tumor cells or their surrounding neighborhoods (Extended Data Fig. 5d). In glioma, neighborhood-level analysis did not reveal consistent pathway enrichment (Extended Data Fig. 5b).

Overall, despite limitations posed by low cell numbers in this assay, we identified distinct transcriptional profiles and biological pathways associated with 16S signal at cellular and neighborhood levels in individuals with BrM and glioma, consistent with our regional DSP analysis.

### The intratumoral bacterial 16S signals correlate with the oral and gut microbiota

Given the large body of literature on the gut–oral–brain axis^25,26^, we set out to determine whether the identified intratumoral bacterial signals are associated with the related gut or oral microbiome. In our prospective cohort, matched stool (glioma *n* = 65, BrM *n* = 28), saliva (glioma *n* = 109, BrM *n* = 42) and cheek swab (glioma *n* = 112, BrM *n* = 42) samples were collected at the time of craniotomy (Supplementary Table 3) and analyzed using metagenomic shotgun sequencing.

**Fig. 6:**
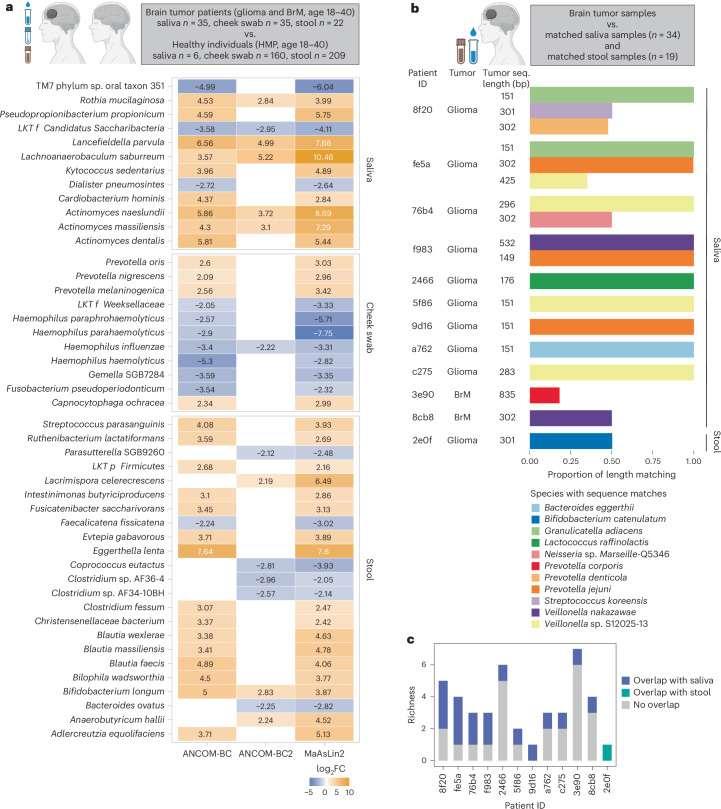
Intratumoral bacterial 16S signal correlates with the oral and gut microbiota of patients with brain tumor. **a**, Differentially abundant taxa in the oral and gut microbiome of patients with glioma and BrM (age range 18–40 years, female 46.7%) compared to healthy individuals (HMP, age range 18–40 years, female 46.4%), assessed by ANCOM-BC, ANCOM-BC2 and MaAsLin2 (two-sided, Holm and Benjamini–Hochberg test for multiple comparison adjustment, respectively). Taxa with differential abundance by at least two methods were included. Heatmap values demonstrate the log_2_FC of taxa with significant differential abundance in patients with brain tumor compared to HMP. If a taxon was not significantly different by two of the three methods, the corresponding cell was colored in white (adjusted *P* values are presented in Supplementary Table 7). Schematic was created using BioRender.com. **b**, The proportion of the identified intratumoral bacterial sequence (seq.) length that overlapped with bacterial sequences in matched oral or gut microbiome (metagenomic shotgun sequencing). Schematic was created using BioRender.com. **c**, Stacked bar plot demonstrating the number of identified taxa in tumor with or without sequence overlap with salivary or stool bacterial taxa.

The gut and oral microbiome in individuals with glioma and BrM did not differ by beta diversity (Bray–Curtis index; stool, *P* = 0.45; saliva, *P* = 0.06; cheek swab, *P* = 0.12), suggesting no correlation with brain tumor type. We then compared the combined glioma and BrM datasets to metagenomic sequencing data from healthy individuals in the Human Microbiome Project (HMP)^27,28^ (stool *n* = 209, *n* = 6, cheek swab *n* = 160). Beta diversity differed significantly between brain tumor-associated and healthy microbiomes (stool and cheek swab, *P* = 0.001; saliva, *P* = 0.014), and we identified distinct oral and gut bacterial signatures in individuals with brain tumor (Fig. 6a and Supplementary Table 7). To control for age, this comparison was restricted to individuals with brain tumor aged 18–40 years, consistent with the HMP cohort; sex was not a confounding factor (stool and cheek swab, *P* > 0.9; saliva, *P* = 0.4). Interestingly, we found that several brain tumor-associated oral and gut bacterial taxa overlapped with the intratumoral bacterial taxa, identified using either 16S amplicon or metagenomic sequencing (*Prevotella*, *Capnocytophaga*, *Streptococcus* and *Bifidobacterium*) (Fig. 6a), suggesting a potential correlation between the intratumoral bacterial signal and the oral and gut microbiota. It should be noted that this comparison may be limited by the differences in methodologies for sample collection and sequencing between the HMP and brain tumor cohorts.

To further evaluate this correlation, we conducted metagenomic shotgun sequencing on 34 matched tumor and saliva samples (glioma *n* = 28, BrM *n* = 6) and 19 matched tumor and stool samples (glioma *n* = 16, BrM *n* = 3) at a sequencing depth of 100 million reads per sample. To avoid cross-contamination, tumor, saliva and stool samples were processed and sequenced separately. In 11 of 34 tumor–saliva sample pairs, we found overlap between the tumor bacterial signal and salivary microbiome, ranging between one and three species per patient, including *Prevotella*, *Veillonella*, *Neisseria*, *Streptococcus* and *Bifidobacterium* species, among others. On average, 79% of the full length of the sequences detected in tumor had overlap with salivary bacterial sequences (percent length matching range 20–100%; tumor sequence length average 292 bp, range 149–835 bp) (Fig. 6b). In tumor–stool sample pairs, sequence overlap was detected in one of 19 tumor–stool sample pairs (*Bifidobacterium*; 50% length matching, tumor sequence length 302 bp). On average, 53% of the total taxa identified in tumors overlapped with either salivary or stool taxa (range 16.6–100%; Fig. 6c). Overall, these findings demonstrate sequence similarity at species level between the oral and gut microbiota and intratumoral bacterial signals.

In addition to distant commensal microbiota, disseminating tumor cells migrating from the primary tumor to the metastatic site may represent another potential source of bacterial signal in BrM. To assess this, we conducted metagenomic shotgun sequencing on a retrospective cohort of matched BrM and primary tumors (*n* = 8 pairs, 50% female, age 47–74 years). After filtration, bacterial taxa were detected in three of eight primary tumors and in two of eight BrM samples; however, no overlapping taxa were identified between matched pairs (Supplementary Table 8).

**Extended Data Fig. 6:**
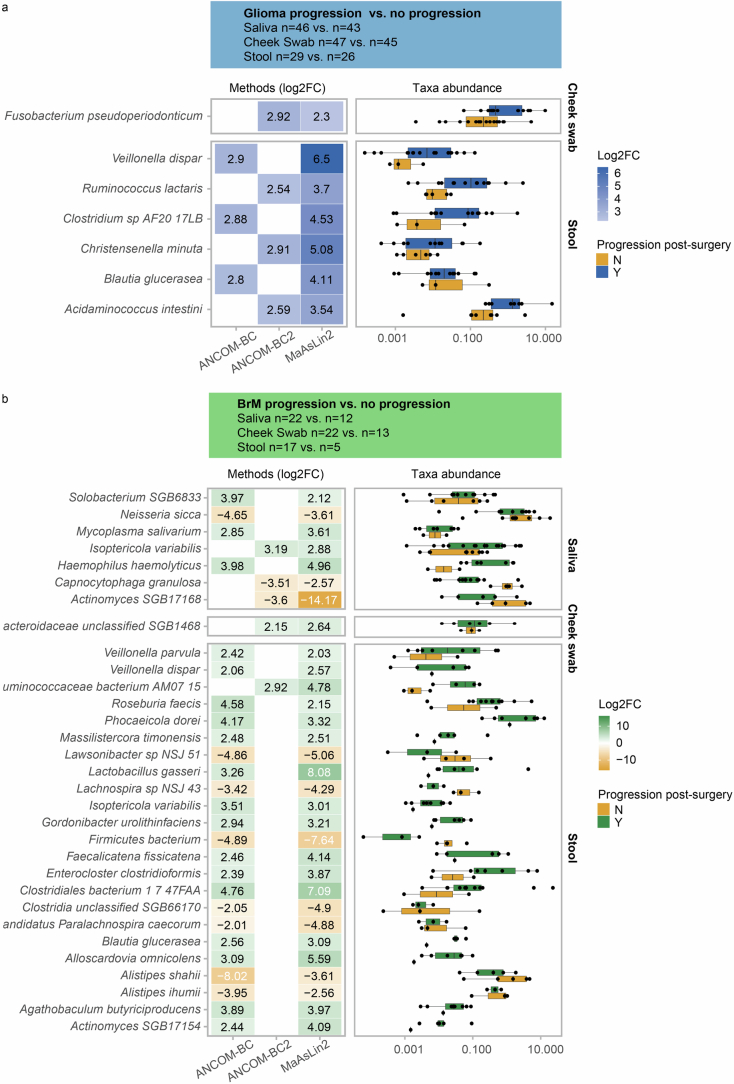
Oral and gut bacterial signatures are associated with brain tumor progression. **a,b**, Differentially abundant taxa in the oral and gut microbiome of (**a**) glioma patients and (**b**) BrM patients with post-resection intracranial progression compared to patients with no progression, assessed by ANCOM-BC, ANCOM-BC2, and MaAsLin2 (two-sided; Holm and Benjamini-Hochberg test for multiple comparison adjustment, respectively). Taxa with adjusted p value < 0.05, log2FC > 2 and with differential abundance by at least two methods are presented. Heatmap demonstrates the log2FC of each taxon with significant differential abundance. If a taxon was not significantly different by 2 of the 3 methods, the corresponding cell was colored in white (adjusted p values are presented in Supplementary Table 9). Boxplots show median (center line), interquartile range (box bounds, 25^th^ and 75^th^ percentiles), and smallest and largest values within 1.5× the IQR (whiskers).

Next, we investigated whether oral and gut microbiota are associated with clinical outcome in individuals with brain tumor (Extended Data Fig. 6). Distinct oral and gut bacterial signatures correlated with intracranial progression post-resection. Specifically, these oral and gut signatures included taxa also detected within the tumor, such as *Fusobacterium* and *Veillonella* (Extended Data Fig. 6 and Supplementary Table 9). This analysis was adjusted for clinical criteria with disproportionate distribution (Supplementary Table 10 and Methods). We also assessed whether the presence or absence of bacterial 16S signal in brain tumors was associated with clinical metrics. Within the limitations of this clinically heterogeneous cohort, intratumoral 16S signal did not independently correlate with patient or tumor characteristics or clinical outcome (Supplementary Table 10).

## Discussion

Malignant brain tumors, particularly GBM and BrM, remain a clinical challenge with limited treatment options^1^. In this study, we identified intracellular bacterial 16S signals as a component of the brain TME, albeit in a subset of tumor samples and at low abundance. Our findings align with recent reports of bacterial nucleic acids in various cancer types, including GBM and CNS metastases^4,7,15,16^, and their heterogeneous distribution within the TME^7^.

Although microbiome studies have historically relied on sequencing and computational analyses, recent reports have raised concerns about the reliability of these standard approaches in low-abundance samples such as tumor tissue^11–13^. This technical limitation was evident in our 16S amplicon and metagenomic shotgun sequencing data analyses, where rigorous decontamination efforts failed to eliminate all known contaminants. As a result, complementary approaches are needed to assess microbial presence in samples with anticipated low abundance. This is exemplified in several studies that used targeted methods to stain bacteria in situ^4,7^, recover cultivatable bacteria from colorectal^6,10^ and melanoma^9^ tumors and detect bacterial activity in breast cancer specimens^4^.

In the present study, we verified the presence and distribution of intracellular bacterial elements using RNA-based FISH, LPS staining and SMI and assessed their biological relevance in the brain TME using spatial technologies. Notably, 16S rRNA FISH, which has been correlated with bacterial size and morphology^29^, revealed signals ranging from small puncta to bacteria-sized structures. Along with LPS detection, these findings suggest the potential presence of both intact and fragmented bacteria. However, our attempts to culture bacteria from brain tumor specimens did not yield any bacterial colonies, likely reflecting the technical challenges of cultivating low-abundance and heterogeneously distributed bacteria from tissue specimens or phenotypic variations of intratumoral bacteria in complex environments that can render them dormant and not cultivable^30^. Nevertheless, the evidence remains insufficient to conclude the presence of a diverse and active microbiota in brain tumors. These findings underscore that the nature and function of bacterial elements within the TME may vary by tumor type and anatomical location, ranging from diverse microbiota in some to low-abundance bacterial elements in others.

The intracellular distribution of bacterial elements observed in our study aligns with previous reports in colorectal and oral squamous cell cancers^6,7^. The intratumoral bacterial taxa that we identified were predominantly Gram-negative intracellular anaerobes (for example, *Prevotella*, *Fusobacterium* and *Capnocytophaga*) as well as facultative intracellular (for example, *Neisseria*) and extracellular (for example, *Gemella*) taxa. Beyond direct internalization or invasion, bacterial elements from both intracellular and extracellular taxa can also enter non-phagocytic cells via bacterial extracellular vesicles, which can carry a variety of biologically active molecules, including proteins, LPS or lipoteichoic acid and nucleic acids (for example, small RNAs and DNA)^31^.

Once internalized, bacteria or their extracellular vesicles can modulate host responses, such as promoting pro-inflammatory signaling (for example, via NF-κB activation) and immune evasion^7,32,33^. Leveraging spatial technologies, we found that intratumoral bacterial signals were associated with antimicrobial, immune and metabolic signatures across cellular, neighborhood and regional levels of the TME. In BrM specifically, components of the TLR9 and NF-κB pathway—an innate immune response to intracellular microbial nucleic acids^19^—were enriched. Additionally, intratumoral 16S signals correlated with increased neutrophil presence and activity, consistent with previous findings in colorectal cancer^7^. Interestingly, 16S-high regions in glioma, but not BrM, showed upregulation of chromatin remodeling proteins, suggesting distinct host responses to bacterial signals in primary versus metastatic brain tumors. Collectively, these data suggest a potential biological role for intratumoral 16S signals in the brain TME. However, owing to the clinical design of this study, causality cannot be established. Instead, our findings provide a foundation for future mechanistic studies to determine the functional consequences of intratumoral bacterial signals.

The origin of the tumor-associated microbial elements, particularly in tumors anatomically distant to the gut microbiota, remains largely unknown. Several mechanisms have been proposed for the transfer of microorganisms and microbial elements to the TME, including colonization by local commensals, hematogenous spread, tumor-mediated and immune-cell-mediated translocation and bacterial extracellular vesicles^6,10,34,35^. In this study, we demonstrated that intratumoral 16S signals overlapped with the oral and gut microbiota at taxa and sequence levels. Interestingly, oral bacterial nucleic acids and proteases, as well as gut bacterial peptides, have been detected in the brains of individuals with neurodegenerative diseases and primary brain tumors^16,36^. These findings suggest a potential mechanistic overlap in the contribution of distant microorganisms to both pathologies. Notably, in this study, a greater number of oral bacteria taxa, compared to gut bacteria, were found to overlap with intratumoral bacterial signal, potentially reflecting the anatomical proximity of the oral cavity and brain as well as shared draining lymph nodes^37,38^. Additionally, the olfactory route has been suggested as a potential path for the transfer of oral microorganisms to the brain in Alzheimer’s disease^39^. Nevertheless, our study also revealed evidence of gut–oral bacterial translocation, with enrichment of oral taxa in the gut microbiota of individuals with brain tumor, and vice versa. This bidirectional exchange, previously reported in different pathologies including cancer^40,41^, may underlie the observed sequence overlaps.

Another possible mechanism, particularly in BrM tumors, is the transfer of bacterial genetic material via circulating metastatic tumor and immune cells, as previously reported in colorectal cancer liver metastases^6^. In a retrospective cohort of matched BrM and primary tumors, we found no correlation between intratumoral bacterial signals in BrM and their corresponding primaries. Although limited by small sample size, this finding suggests that tumor cell dissemination may not be the predominant route of bacterial signal transfer. The presence of bacterial signals in non-metastatic brain tumors and their distribution across brain-resident stromal cells further supports this notion. Nevertheless, several bacterial taxa identified here (for example, *Fusobacterium nucleatum*) have also been found in other solid primary tumors, underscoring the need for mechanistic studies using bacterial tracing to rigorously evaluate these relationships. Collectively, our findings highlight a complex interplay among intratumoral bacterial elements, the brain TME and distant microbial communities.

The key limitations of this study arise mainly from the clinical setting and the correlational nature of the analyses as well as the modest sample size with respect to cohort heterogeneity, hindering our ability to comprehensively assess the biological and clinical significance of the intratumoral bacterial signals. Insights gained from this endeavor can guide future studies to address important gaps in the field, such as determining the mechanisms driving the transfer of 16S signal to brain tumors, their causal role in shaping the TME and their impact on brain tumor progression. Lastly, microbiota composition can be heavily affected by geographical, environmental and lifestyle factors^42,43^. Therefore, discerning the universality of bacterial taxa linked to brain tumors, whether intratumoral or distant, and their individual and collective clinical impact will require standardized multi-institutional investigations across diverse populations and geographic locations. We advocate for collaborative efforts in the field to accomplish these goals.

## Methods

### Human study participants

#### MDACC cohorts

Patients with primary and metastatic brain tumors undergoing surgical resection of brain lesions were enrolled in this study. Under the CNS Tumor AnaLYsis STream (CATALYST) program, written informed consent was obtained from all patients during their pre-surgery appointment at MDACC or inpatient. All samples were collected under approved Institutional Review Board (IRB) protocol (2012-4041), and in accordance with the 1964 Declaration of Helsinki and its later amendments.

#### UTH cohort

Patients with metastatic brain tumors were prospectively enrolled to collect tumor samples for microbiome analysis. Non-cancerous brain samples were collected as part of resective epilepsy procedure for medically refractory epilepsy. Patients provided written informed consent for sample collection under the IRB-approved protocol (HSC-MS-0967) and in accordance with the 1964 Declaration of Helsinki and its later amendments.

Demographic information, including age, sex (self-report) and race, as well as clinical and sample-related information collected from medical records, are reported in Supplementary Table 3 and, when necessary, were considered as confounding factors. Information on gender was not available in the medical records and, therefore, was not collected for this study.

### Sample collection

#### MDACC cohorts

MDACC1 cohort (glioma and BrM): Tumor samples were collected sterilely in the operating room and snap frozen in liquid nitrogen under sterile conditions in the laboratory. Patients were provided an OMNIgene·GUT kit (OMR-200), an OMNIgene·ORAL kit (saliva: OM505, cheek swab: OMR-120) (DNA Genotek) and instructions for sample collection. Cheek swab and saliva samples were collected in clinic. Fecal samples were either collected in the clinic or mailed back to the clinic after patient discharge. Upon receipt in the laboratory, samples were aliquoted and stored at −80 °C.

MDACC2 (glioma): Tumor samples available for distribution to research laboratories at the institutional biobank were requested for microbiome analysis. Samples included fresh-frozen tumor tissue, collected as described above, FFPE tissue (*n* = 5) and OCT-embedded tissue (*n* = 4). For FFPE and OCT samples, a punch from each block was included as a sample control.

#### UTH cohort

Tissue samples from the patients were directly placed by the surgical team in sterile pre-labeled cryotubes or a sterile recipient and then transferred to cryotubes in strict sterile conditions. The cryotubes were snap frozen in liquid nitrogen immediately after being obtained and transferred to storage until microbiome analysis in a −80 °C freezer. Fresh-frozen non-cancerous brain tissue was received from an archived biospecimen repository.

### Clinical data collection

Electronic medical records were reviewed by a neurosurgeon who was blinded to the microbiome analysis results. Demographic information, including age at the time of surgery, sex and race, was retrieved. For patients with glioma, histopathology, grade and IDH mutation status and recurrence status of the tumor at the time of surgery (that is, whether the tumor was a treatment-naive/newly diagnosed (primary) or a recurrent lesion at the time of surgery) were recorded. Patients who underwent a previous biopsy at an outside institution (prior to presenting to MDACC) but received no other therapy were categorized as treatment naive/newly diagnosed. For patients with BrM, histopathology findings, primary tumor of origin/systemic cancer and history of previous treatments, including cranial radiation (for example, stereotactic radiosurgery and whole-brain radiation therapy), immunotherapy and targeted therapy, as well as status of leptomeningeal disease and systemic (extracranial) disease, were collected. Systemic disease status was determined from review of computed tomography scan of the chest/abdomen/pelvis or a body positron emission tomography scan. In the case of body imaging studies that were performed outside of MDACC without an accompanying radiology report, systemic status was classified as ‘unknown’. Uncontrolled systemic disease was defined as disease that showed progression compared to previous surveillance imaging and/or development of new metastatic lesion; controlled disease showed no radiographic progression. For both glioma and BrM, multifocality and anatomical location of the resected tumors, preoperative steroid treatment and history of systemic or intracranial bacterial infection were recorded. For cases with positive history of bacterial infection, when available, time of infection from tumor resection, the involved organism and antibiotic treatment were collected. Tumor size (diameter of the lesion) at the time of surgery was recorded based on magnetic resonance imaging (MRI) records. If the lesion was predominantly enhancing, the maximal diameter of the contrast enhancement was recorded. If the lesion was predominantly T2 fluid-attenuated inversion recovery (FLAIR), this sequence was used for measurement. Furthermore, for both glioma and BrM, any evidence of intracranial progression during the post-surgery follow-up, as well as duration to progression and the total post-surgery follow-up time, were recorded. Progression was defined as detection of new intracranial lesions during post-operation follow-up assessed by imaging. Radiographic progression had to be documented in the associated MRI report. For patients who had additional surgery, progression was further confirmed by histology. In cases of no additional surgery and non-confirmative imaging, patients were considered to have had progression if they received treatment for intracranial lesions. Progression status was marked as ‘unknown’ if patients died before intracranial follow up, and, therefore, the cause of death could not be attributed to intracranial relapse, or if patients did not return to MDACC or UTH for follow up or there were no verifiable imaging reports documenting radiographic progression.

### Microbiome extraction and sequencing methods

All samples were processed for DNA extraction and sequenced at CosmosID, Inc. or the MDACC Microbiome Core Facility (Supplementary Table 3). Fresh-frozen tumor samples were submitted for DNA extraction and sequencing without further thawing or aliquoting in the laboratory to minimize contamination. For FFPE and OCT-embedded samples, a paraffin/OCT punch was included as sample control. Reagent controls were taken at the time of DNA extraction for each extraction kit and each batch and at the time of library preparations. All tumor samples and associated controls were sequenced using V3-V4 16S rRNA amplicon sequencing. A subgroup of sample and controls was sequenced via metagenomic shotgun sequencing. Matched stool (approximately 100 mg), saliva and cheek swab (500 µl each) samples were sequenced using metagenomic shotgun sequencing at 30 million reads. A subgroup of stool and saliva samples was also sequenced at 100 million reads.

#### Bacterial DNA extraction

DNA extraction from fresh-frozen or OCT-embedded tumor samples was conducted with a DNeasy UltraClean Microbial Kit (Qiagen). DNA extraction from FFPE samples was conducted with a QIAamp DNA FFPE Tissue Kit (Qiagen). DNA extraction from stool, cheek swab and saliva samples was conducted using a Qiagen DNeasy PowerSoil Pro Kit. The manufacturer’s instructions were followed for all DNA extractions. Genomic DNA was quantified with Qubit 2.0 DNA HS Assay (Thermo Fisher Scientific). All tumor samples were processed separately from oral and stool samples to avoid cross-contamination.

#### 16S rRNA amplicon sequencing

Genomic DNA was amplified via PCR using the following primers: 341F (CCTACGGGRSGCAGCA) and 805R (GACTACHVGGGTATCTAATCC)^44^, which cover hypervariable regions (V3 and V4). Primer selection and design were chosen to achieve comprehensive taxonomic coverage. Final library quantity was assessed by Qubit 2.0 (Thermo Fisher Scientific), and quality was assessed by TapeStation D1000 ScreenTape (Agilent Technologies). Illumina 8-nucleotide dual-indices were used. Equimolar pooling of libraries was performed based on quality control values, and samples were sequenced on an Illumina MiSeq with a read length configuration of 300 bp for 0.1 million paired-end reads per sample (500,000 in each direction).

#### Metagenomic shotgun sequencing

DNA libraries were prepared using a Nextera XT DNA Library Preparation Kit (Illumina) and IDT unique dual indexes with total DNA input of 1 ng. Genomic DNA was fragmented using a proportional amount of Illumina Nextera XT fragmentation enzyme. Unique dual indexes were added to each sample followed by 12 cycles of PCR to construct libraries. DNA libraries were purified using AMPure magnetic beads (Beckman Coulter) and eluted in Qiagen EB buffer. DNA libraries were quantified using a Qubit 4 fluorometer and a Qubit dsDNA HS Assay Kit. Libraries were then sequenced on an Illumina NovaSeq S4 platform at 2 × 150 bp. Sequencing for tumor and matched saliva and stool samples was conducted at 100 million reads per sample and for saliva, cheek swab and stool samples at 30 million or 100 million reads per sample.

### Microbiome analytical pipelines

#### Tumor 16S rRNA amplicon sequencing

16S rRNA sequencing datasets (MDACC1, MDACC2, UTH tumor and non-cancerous brain cohorts) were analyzed using the DADA2 package (version 1.26)^45^. All taxonomic output files were imported into R using the phyloseq package (version 1.50.0)^46^. To eliminate signal from potential contaminants, we used a five-step filtration process:

Step 1. To eliminate contaminants introduced by reagents and operators (expected to be in high abundance and across most samples), we pooled all controls across all cohorts including DNA extraction, library preparation and blank controls as well as FFPE and OCT punch controls taken from each block (*n* = 24) and plotted the distribution of ASVs based on proportion across all controls (Extended Data Fig. 3a). Using the changepoint R package (version 2.3), we calculated the first detectable inflection point on this curve, which was found to be 0.045%. We used this threshold to eliminate sample ASVs that follow a similar distribution to those of controls, and only ASVs with less than 0.045% proportion across all samples were kept for the next filtration step.

Step 2. Using the same approach, we plotted the distribution of genera based on prevalence across all controls (Extended Data Fig. 3a) and determined the inflection point at 8.3%. Accordingly, all genera that were prevalent in more than 8.3% of samples were removed.

Step 3. To reduce batch bias, we excluded all genera that were present exclusively in samples from a single batch.

Step 4. We used Source tracking for Contamination Removal in microBiomes (SCRuB, version 0.0.1)^47^, using the well position for samples in each cohort, to eliminate ASVs that could be a result of potential cross-contamination across samples that were sequenced together.

Step 5. We removed any remaining ASVs identified in batch-specific controls from samples in the relevant batch.

The identified taxa after these filtration steps were plotted using ggplot2 (version 3.5.2)^48^. Specifically, we highlighted taxa that were linked to common human commensal microorganisms and those that are environmental contaminant, separately, for ease of review. Alluvial plots demonstrating the removal of taxa across the different filtration steps were generated using ggalluvial (version 0.12.5).

#### Tumor metagenomic shotgun sequencing

Metagenomic shotgun sequencing data from all tumor samples as well as matched saliva and stool samples (100 million) were analyzed using a custom pipeline for the detection of reads of bacterial origin. Single and paired-end reads were quality trimmed and quality filtered using BBDuk. Reads of human origin were filtered using the bloom filter from the BBTools suite^49^ using a cutoff of more than 15 31mers matching the human genome (GRChg38)^50^. Paired-end sequencing reads were merged using BBMerge from the BBTools suite^49^ under ‘maxstrict’ parameters. Combined merged and single-end reads were dereplicated using VSEARCH^51^. Dereplicated reads were normalized using normalized-by-median.py from the khmer suite^52^. Normalized reads were filtered against a bloom filter containing all kmers from gbbct, and single kmer match was enough to allow the read to pass. The resulting read set was mapped against all bacterial reference entries in GenBank bacteria (gbbct) using BLASTn^53^ with an E-value cutoff of 0.00001, a word length of 29 and a 95% identity cutoff. All aligned reads were extracted and aligned to SILVA^17^ 16S database version 138. Reads aligning to 16S were removed. Remaining reads were clustered using a minimum set cover approach to determine which minimal set of genomes contained all remaining read alignments.

For tumor samples, aligned bacterial reads were aggregated into a single bulk sample. The reads were subsequently remapped to all bacterial genomes in gbbct. Reads with top-scoring hits that contained more than one genus, or two distinct species, were considered to be of low informativity and were removed from consideration. The resulting read set was reclustered using a similar minimum set cover approach to determine which genomes best explained the highly informative reads. This clustering step also included querying whether a genome contained reported assembly anomalies or was derived from metagenome-assembled genomes (MAGs), which were removed. The resulting set of genomes was considered to be our basal set (Step 1). To further remove potential contaminants, we used additional filtration steps:

Step 2. Taxa identified in negative controls were removed as contaminants.

Step 3. Hits smaller than the median contig length (139-bp length) were removed as low-abundance contaminants.

Step 4. Depth of coverage per species was calculated, and unique sequences for which more than two times coverage was detected across all tumor samples were removed as high-abundance/high-redundancy contaminants.

#### Comparison of bacterial sequences in tumor versus matched saliva and stool

To assess sequence overlaps among tumor, saliva and stool, the set of bacterial genomes reconstructed in tumors was used to reconstruct bacterial information from saliva and stool sequencing reads on a per-sample basis. The resulting reconstructions were aligned to each other to determine overlap of species across sample types at an overlap threshold of more than 95%. If the same species occurred in different samples from the same patient at more than 98% identity, it was considered to be the same genomes. Overlapping segments between samples of the same patient were compared to the same determined species in samples from other patients at more than 95% identity to exclude segments with similar or higher overlap among non-matched samples.

#### Comparison of bacterial sequences in tumor versus matched saliva and stool

Metagenomic shotgun sequencing (stool, saliva and cheek swab samples from the MDACC1 cohort, 30 million) were filtered and mapped using BBMap (version 38.84) and analyzed using the MetaPhlAn package (version 4.1.1.)^54^ and the CHOCOPhlAn database (mpa_vJan21_CHOCOPhlAnSGB_202103).

#### Analysis of the microbial differential abundance

Differential abundance of bacterial taxa in saliva, cheek swab and stool samples was evaluated by three methods, including Microbiome Multivariable Association with Linear Models (MaAsLin2, version 1.20.0)^55^ and Analysis of Compositions of Microbiomes with Bias Correction (ANCOM-BC and ANCOM-BC2 functions within package version 2.8.1, which account for sample-specific and taxon-specific bias, respectively)^56,57^. Taxa that were identified to be differentially abundant by at least two methods were included for plotting. For all methods, taxa with more than 25% prevalence among all samples and a minimum abundance of 100 reads were selected. Default setting was used for all methods except for struc-zero and neg-lb for ANCOM-BC and ANCOM-BC2, which were set as TRUE and analysis_method = ‘NEGBIN’ and transform = ‘NONE’ and normalization = ‘TMM’ for MaAsLin2. To identify confounding factors, the distribution of different clinical criteria was evaluated by Pearson’s chi-squared test with ‘simulate.p.value = TRUE’ or Wilcoxon rank-sum exact test. For post-surgery progression as the main predictor, these covariates included sequencing batch for BrM saliva and cheek swab; age, grade and IDG mutation for glioma saliva, cheek swab and stool; and steroids at surgery for glioma stool. The differential abundance analyses were controlled for those significant. Plots were generated for taxa with an adjusted *P* < 0.05 and a log_2_FC > 2, using ggplot2.

#### dPCR

dPCR was conducted using the 16S pan-bacterial assay (TaqMan, Ba04930791-S1; Thermor Fisher Scientific) and the QuantStudio Absolute Q dPCR System (Thermo Fisher Scientific, A52732 and A53301), following the manufacturer’s instructions. Nuclease-free water was used as a negative control.

#### TMAs

A total of 33 glioma and 19 BrM FFPE blocks were used for TMA construction. Hematoxylin and eosin (H&E) slides of each block were used for initial screening to demarcate immune-high and immune-low areas in the tumor. For each block, 2–6 cores were selected for TMA construction. TMAs were built using an ATA-100 Advanced Tissue Arrayer (Chemicon International). A total of 103 and 96 cores, 1 mm in diameter each, were taken from glioma and BrM blocks, respectively. The cores were arranged to fit within a 36.2-mm × 14.6-mm rectangle, as required by the GeoMx Digital Spatial Profiler (Bruker Spatial Biology). For sectioning, all surfaces were cleaned by bleach, and new blades and fresh reagents were used each time. Five-micrometer fresh-cut sections of the glioma and BrM TMAs were mounted onto Superfrost Plus slides and were used for RNAScope FISH, SMI and DSP experiments. Fresh-cut normal brain TMA sections were purchased from TissueArray (BNC17011d) for RNAScope FISH. To minimize contamination, TMA core needles were cleaned with DNA AWAY (Thermo Fisher Scientific) in between each core. For slide sectioning, the histotech’s water bath was wiped with DNA AWAY and refilled with deionized water. The block surfaces and cutting tools were wiped with DNA AWAY. A new microtome blade was used for each sample and wiped with DNA AWAY prior to use. Five-millimeter-thick sections were cut and left to dry in a sterile biocabinet and subsequently stored at 4 °C in a slide box whose inner surfaces had been wiped with DNA AWAY.

#### RNAScope FISH

To visualize bacterial 16S signals within the TME, glioma, BrM and healthy brain TMA or whole-tissue sections were stained using the RNAScope Multiplex Fluorescent Assay (RNAScope Multiplex Fluorescent Reagent Kit version 2, 323100; Advanced Cell Diagnostics). In brief, slides were baked at 60 °C for 1 hour using a Quincy 20GC analog lab oven and deparaffinized using xylene and 100% EtOH (2×). Slides were then treated with hydrogen peroxide for 10 minutes at room temperature. Antigen retrieval was conducted using RNAScope 1× Target Retrieval reagent within a steamer at 99 °C for 15 minutes. The slides were washed with 100% EtOH and dried at room temperature overnight. On the second day, the slides were treated with RNAScope Protease Plus at 40 °C for 20 minutes using a hybridization chamber (Boekel Scientific RapidFISH Slide Hybridizer, 240200). Each slide was then incubated with RNAScope Probe Mix including a eubacteria 16S probe (RNAScope Probe - EB-16S-rRNA-C2, no. 464461-C2) and a negative control that targets (RNAScope Probe - Dr-igf1, no. 489251) at 40 °C for 2 hours. Amplification was conducted through three rounds of incubation with RNAScope Amplification Buffers, followed by separate channel development for each probe (eubacteria 16S, cyanine 3; negative control probe, cyanine 5). For whole-tissue sections co-stained with pan-cytokeratin or GFAP, protease incubation was performed for 15 minutes. Serial sections were incubated with either 16S probe or negative control probe (RNAScope Probe - Dr-actb2, no. 486831-C2). Channel development was conducted for both 16S and negative control probes with cyanine 5 (Akoya Biosciences, NEL745001KT, 1:3,000). Slides were fixed with 10% neutral buffered formalin (NBF) and then stored in 1× PBS at 4 °C overnight. The next day, slides were blocked with Buffer W for 30 minutes at room temperature and then stained with antibody diluted in Buffer W for 1 hour at room temperature. Glioma and BrM samples were stained with anti-GFAP (Alexa 388-GFAP, eBioscience, 53-9892-82, 1:50) and PanCK (Alexa 532-PanCK, Novus Biologicals, NBP2-33200AF532, 1:50), respectively. Slides were washed with 2× saline-sodium citrate (SSC) buffer for 5 minutes at room temperature. To minimize autofluorescence from lipofuscin, which can be detected in brain tissue with aging^58^, slides were treated with 1× TrueBlack Lipofuscin Autofluorescence Quencher (Cell Signaling Technology, 92401) for 30 seconds at room temperature, followed by three rounds of PBS washes. Slides were then incubated with DAPI for 30 seconds at room temperature and mounted using ProLong Gold Antifade Mounting Solution.

Imaging of the 16S-stained tissue sections was conducted using the GeoMx platform (Bruker Spatial Biology). High-magnification imaging was conducted using a Zeiss Apotome 3 microscope and at ×20 dry and ×63 oil objectives. Exposure time was set at 60 ms for DAPI and 3 seconds for 16S and negative control channels. *z*-stack images were captured of 16S-positive areas at ×63 objective, and three-dimensional renderings were created using Zeiss ZEN software (version 3.9.).

#### Bacterial culture

Tumor samples were cultured on four types of media:

- Difco tryptic soy agar/broth (DF0369-17-6) with sheep blood (TSA/B-SB, aerobic and anaerobic)
- Fastidious anaerobe agar (50-201-5192) with sheep blood (FFA-SB, anaerobic)
- Chocolate agar (R01300) (anaerobic)
- A specialized and enriched nutritious growth medium (BYESRF, aerobic and anaerobic) was formulated, comprising BHI (agar and broth) supplemented with 5 g of yeast extract (Thermo Fisher Scientific, DF0127-17-9), 5% sterilized rumen fluid (Thermo Fisher Scientific, NC1530570), vitamin supplements (American Type Culture Collection (ATCC), MD-VS), trace mineral supplement (ATCC, MD-TMS), 10 µg ml^−1^ hemin (Sigma-Aldrich, 51280-5G), 10 µg ml^−1^ vitamin K3 (Sigma-Aldrich, M5625), 5 mg ml^−1^ L-cysteine (Sigma-Aldrich, 168149-100G) and a mixture of monosaccharides and disaccharides (500 mg each), including fucose (Sigma-Aldrich, F2252), arabinose (Sigma-Aldrich, A3131), galactose (Sigma-Aldrich, G0625), fructose (Sigma-Aldrich, 05323.0250), ribose (Sigma-Aldrich, R1757), mannose (Sigma-Aldrich, M8574), xylose (Sigma-Aldrich, X3877), cellobiose (Sigma-Aldrich, C7252) and maltose (Sigma-Aldrich, M5885).

A total of nine fresh tumor samples were obtained from the operating room and were transferred to a tube containing 500 µl of autoclaved TSA medium, under a biological safety cabinet. A second tube of autoclaved TSA was opened under the hood, at the time of transfer, to serve as an environmental control. In addition to fresh tumor, 10 fresh-frozen tumor samples and two fresh-frozen non-cancerous brain samples (temporal lobe) were also included for culture. In an anaerobic chamber (Whitley A135 HEPA Anaerobic Workstation; Don Whitley Scientific), one 5-mm stainless steel bead (Qiagen, 69989) was added to the tubes for homogenization via a TissueLyzer LT (Qiagen, 85600) at 50 Hz for 5 minutes. For each media type, 50 µl of the tissue homogenates was spread on 60-mm plates using plating beads. Furthermore, 50 µl of samples was added to the TSB-SB broth media. Cultures were kept in anerobic and aerobic conditions at 37 °C and evaluated for colony formation every day for 14 days. Broth samples were cultured on agar plates after 72 hours and continued for another 14 days with routine evaluation for colony formation. For each new batch of media, bacterial taxa, including *Rothia mucilaginosa* (ATCC, 25296) and *Prevotella intermedia* (ATCC, 25611), were used as positive controls. Colony biotyping was conducted using a MALDI Biotyper (Bruker).

Three fresh-frozen samples were cultured independently in the laboratories of S.B. and C.D.J. In brief, tissue sections were minced with a scalpel and spread plated on two media types: selective FAA plates (Neogen) supplemented with 10% defibrinated sheep blood (DSB) (Innovative Research) and chocolate agar with FAA (Neogen) supplemented with 10% DSB, warmed at 80 °C, as a base medium. Plates were incubated at 37 °C in both aerobic and anaerobic conditions (Oxoid AnaeroGen gas generating system; Thermo Fisher Scientific) and inspected for growth every 24 hours and 48 hours, respectively.

All frozen samples were confirmed to contain bacterial reads via SMI, 16S amplicon and/or metagenomic shotgun sequencing. Fresh samples were evaluated for bacterial presence when sufficient tissue was available for 16S rRNA dPCR.

#### IHC

After treating all FFPE block and microtome surfaces with DNA AWAY to remove exogenous microbial biomolecules, samples were sectioned at 4 µm onto Superfrost Plus slides (Thermo Fisher Scientific). IHC staining was performed using a BOND RXm autostainer (Leica). Slides were subjected to a 20-minute antigen retrieval in BOND pH 6 citrate buffer (Leica) at 100 °C. Mouse monoclonal antibody against LPS lipid A (Abcam, ab8467) was diluted 1:500 in BOND primary antibody diluent (Leica) and applied to the slides for 15 minutes at room temperature. Staining proceeded using the BOND Polymer Refine Detection system using a hematoxylin counterstain. A colon section containing fecal material was used as a positive control. IHC slides were scanned at ×20 using an Aperio AT2 whole-slide scanner (Leica) and assessed using ImageScope software. Images were assessed for LPS staining by a pathologist.

Multiplex fluorescence staining was conducted using the Leica BOND RX autostainer and the Opal 7-color kit (Akoya Biosciences, NEL871001KT). Two separate lymphoid and myeloid panels were used for staining both glioma and BrM TMAs. The following antibodies were included in the lymphoid panel: CD8 (1:200; Novus Biologicals, NBP2-29475), CD4 (1:1,000; Abcam, 133616), GZMB (1:100; Cell Signaling Technology, 46890), FoxP3 (1:50; Cell Signaling Technology, 98377), CD16 (1:12,000; Invitrogen, Pa5-80622) and CD56 (1:50; Cell Signaling Technology, 99746). The following antibodies were included in the myeloid panel: IBA1 (1:8,000; Abcam, 178847), CD163 (1:500; Abcam, 182422), CD206 (1:4,000, Abcam, 64693), Arginase 1 (1:4,500, Invitrogen, PA5-85267), CD11b (1:6,000; Abcam, 133357) and CD11c (1:400; Cell Signaling Technology, 45581). Whole-slide imaging was performed at ×20 magnification using an Aperio Versa Pathology Scanner 8 (Leica). Images were analyzed by a pathologist and captured using HALO image analysis software (Indica Labs, 3.6.4134.166). Image brightness and contrast were adjusted in ImageJ. Positive cell counts were log_2_ transformed, and significance was evaluated using an LMM and the Benjamini–Hochberg procedure for multiple hypothesis testing correction.

### SMI (CosMx platform)

Spatial molecular imaging was conducted using the CosMx SMI platform (Bruker Spatial Biology) using the 6K Discovery panel and a custom panel of bacterial probes. This experiment was performed in collaboration with Bruker Spatial Biology.

#### Bacterial probes

The 6K human transcriptome panel was supplemented with a panel of pan-bacterial 16S probes as well as genus-specific probes against *Porphyromonas*, *Fusobacterium* and *Prevotella* (five probes each; Supplementary Table 11). Bacterial probes were mapped to SILVA^17^, a comprehensive ribosomal RNA sequence database, and demonstrated a hit rate of 86.3% and 72.3% against all available bacterial accessions and taxa, respectively. Bacterial probes were designed and confirmed to have no overlap with human transcripts, including mitochondrial rRNA. Specifically, sequences of all 16S rRNA probes used were mapped against all sequences in GenBank under *Homo sapiens* (taxa ID 9606). This includes GRCh38 (GCF_000001405.26) and T2T (GCF_009914755.1). GRCh38 includes mitochondrial DNA (NC_012920.1). Mapping parameters included a seed size of seven nucleotides, E-value of 10 and 40% query coverage while excluding bacteria and archaea (taxa IDs 2 and 2157, respectively). These parameters were selected to cast a wide net of all potential alignments and purposely allowing noise for further manual evaluation. All but one alignment were found to be either mislabeled bacterial sequences or bacterial segments of bacterial artificial chromosome (BAC) clones. The remaining alignment was observed in the wrong orientation for RNA binding.

#### Sample preparation

Slides were prepared according to the manufacturer’s protocol for FFPE slide preparation. In brief, slides were baked at 60 °C overnight, followed by deparaffinization, proteinase K digestion (3 μg ml^−1^, 40 °C, 30 minutes) and antigen retrieval (100 °C, 15 min, in Leica buffer ER1). Samples were then fixed using 10% NBF, rinsed with Tris-glycine buffer (0.1 M glycine, 0.1 M Tris-base) and blocked using 100 mM N-succinimidyl acetate (NHS-acetate; Thermo Fisher Scientific) in NHS-acetate buffer (0.1 M NaP, 0.1% Tween (pH 8) in diethylpyrocarbonate (DEPC) water) for 15 minutes at room temperature. The slides were then covered with SecureSeal adhesive hybridization chamber (Grace Bio-Labs).

RNA in situ hybridization (ISH) probes (6K Discovery Panel, cat. no. 121500041) were denatured at 95 °C before preparation of the ISH probe mix (1 nM ISH probes, 1× buffer R, 0.1 U μl^−1^ SUPERase·In).

Slides were incubated with ISH probe mix at 37 °C overnight. On the second day, after recommended wash steps, slides were stained with DAPI for 15 minutes and then a cocktail of antibody and ISH probe markers for 1 hour. For glioma, the antibody/ISH probe cocktail included Histone H3 morphology marker (Alexa Fluor 488, AF488, 1:20, lot no. 0519127), 16S rRNA (Alexa Fluor 532), 18S rRNA (Alexa Fluor 594) and GFAP (Alexa Fluor 647, 1:25, lot no. 0519126). For BrM, the cocktail used was CD45/PanCK pooled morphology markers (Alexa Fluor 647/Alexa Fluor 488, 1:25, lot no. LN0519123), 16S rRNA (Alexa fluor 532) and CD298/B2M (Alexa Fluor 594, 1:100, lot no. LN0519122).

#### Spatial transcriptomic measurement

Target RNA readout was conducted following published protocols^59^. After loading on the instrument, the flow cell was scanned, and 39 FOVs were created on each slide. For the first RNA readout cycle, Reporter Pool 1 was flowed into the flow cell, rinsed to remove unbound reporter probes and replaced with imaging buffer. Eight *z*-stack images (0.8-μm step size) were acquired of each FOV, and the fluorophores on the reporter probes were ultraviolet cleaved and washed. This readout step was repeated for the 26 reporter pools and 27 cycles. Hybridization imaging was repeated eight times to increase RNA detection sensitivity.

#### Image processing and cell segmentation

Raw images were processed following published data processing pipelines^59^. For cell segmentation, *z*-stack images of immunostaining and DAPI were used for determining cell boundaries. Transcripts were assigned to cell locations and subcellular compartments using a cell segmentation pipeline and machine learning algorithm^60–62^. The transcript profile of individual cells was generated by combining target transcript location and cell segmentation boundaries. Cells with fewer than 20 total transcripts were removed from the analysis.

#### Cell type annotation

Semi-supervised cell typing was conducted using a negative binomial model with the InSituType package (https://github.com/Nanostring-Biostats/InSituType), using the default settings^63^. A range of 12–20 clusters for BrM and 12–30 clusters for glioma was used for novel cluster discovery, incorporating the fluorescence from the main morphology markers and using the update reference option to update the profile for use with the CosMx dataset. For BrM, the matrix profile from Gonzalez et al.^64^ was used as the main reference, and profiles from the CosMx-based cell human brain and immuno-oncology profiling matrix, as well as Adams et al.^65^, were used as references to assign novel clusters. For glioma, Darmanis et al.^66^ was used as the main reference, and novel clusters were assigned using the same matrix profiles described for BrM. Cell assignments were verified via known marker genes, protein expression when available and location within tissue.

#### 16S-positive cell annotation

First, a cell-specific background level was inferred using the counts from all negative probes, which were synthetized separately for host and bacterial probes but with the same chemistry. Each cell’s estimated background was calculated as a proportion of its total RNA content. To prevent inflation of normalized values in low-RNA cells where background estimates could be near zero, we imposed a lower bound on the estimated background set as the 5th percentile of non-zero background values across the dataset. We then normalized the raw bacterial counts to this cell-specific background level. To identify positive cells, we performed receiver operating characteristic (ROC)-based thresholding, using a subset of confident pseudo-labels, to define a cell with zero microbial counts as a robust negative cell and a cell with normalized counts more than 20 as a robust positive cell. The threshold to call a cell positive for bacterial counts was calculated as 10.01 counts for glioma and 10.02 counts for BrM. To ensure intracellular localization of the 16S signal, cells were annotated as high-confidence 16S-positive if the median 16S position was within the central 50% of all transcript positions across the *x*, *y* and *z* axes (Extended Data Fig. 1d).

To quantify the spatial density of bacterial signal, we computed the number of microbial 16S transcripts per unit area within each FOV. Transcripts were annotated as either intracellular or extracellular based on the presence or absence of a corresponding segmented cell. Intracellular transcripts were mapped to individual cells, and the total intracellular signal per FOV was divided by the sum of the cell areas (in µm^2^) to obtain intracellular density. Extracellular transcripts were defined as those with cell_ID = 0, and their total per FOV was normalized to the extracellular area, calculated by subtracting the total segmented cell area from the fixed FOV area (4,256 × 4,256 pixels × 0.12^2^ µm^2^ per pixel). Densities were expressed as transcripts per µm^2^. Per-FOV intracellular and extracellular densities were summarized by computing the median, minimum and maximum values across all FOVs.

#### Dimensionality reduction

For both glioma and BrM datasets, Pearson residuals from host probes were reduced to 25 principal components using PCA, followed by uniform manifold approximation and projection (UMAP) with a cosine distance metric (20 neighbors, minimum distance = 0.1, spread = 3) to visualize cell relationships in two dimensions.

#### Spatial neighborhood/niche annotation

16S-positive neighborhoods were defined within a 30-μm radius of a 16S-positive tumor cell in two-dimensional physical space.

16S-negative neighborhoods were defined as cells within 30 μm of a negative tumor cell. All cells that were more than 30 μm away from a tumor cell and those within 100 μm of the closest 16S-positive cell were excluded from analysis.

#### Differential expression

To identify transcripts with differential expression between 16S-positive and 16S-negative cells and neighborhoods, a generalized LMM was used, regressing raw counts for each gene against group using the R package NEBULA^67^. The model included an offset term for each cell’s ‘library size’ or total expression across all genes. To enhance neighborhood differential analysis and to bolster the model against potential cell segmentation and cell type uncertainties intrinsic to image-based spatial transcriptomic technologies, the total expression of the analyzed genes in neighboring cells of dissimilar cell type was calculated and included as a fixed effect covariate in the regression model, with patient ID as a random intercept to account for repeated measures.

To determine pathway activity at the cell and neighborhood level, we conducted a targeted search of GOBP gene sets and Reactome pathway database for key terms related to the differentially expressed genes detected by SMI and DSP. Using these curated annotations, we developed custom pathways, reflecting key processes highlighted by DSP (Supplementary Table 12).

Pathways were scored for 16S-positive and 16S-negative tumor cells and neighborhoods, using AUCell^68^. LMM was used to detect differences between 16S-positive and 16S-negative tumor cells and neighborhoods across all patients, with bacterial status as a fixed effect and patient ID as a random intercept to account for repeated measures, using the lmer function from the lmerTest R package. The Benjamini–Hochberg post hoc test was used for multiple hypothesis testing correction.

#### Visualization and three-dimensional cell construction

Visualization of 16S-positive cells was conducted using a NanoString-developed custom plugin for the image software napari (version 0.4.17). Three-dimensional scatter plots of 16S-specific or genus-specific positive cells were generated using the Plotly package (version 5.21.0) in a Python (version 3.9.6) environment. Spatial maps of neighborhoods were created using ggplot.

### DSP (GeoMx platform)

#### Sample preparation

The glioma and BrM TMA slides were deparaffinized and processed using the manual GeoMx spatial proteogenomic assay, which enables simultaneous detection of RNA and protein on the same slide, following the manufacturer’s recommended protocol. Slides were incubated with the GeoMx IPA (cat. no. 121300312) and the 18,000-gene human WTA (cat. no. 121401102) (Bruker Spatial Biology), complemented with a custom panel of pan-microbial markers, including bacterial 16S as previously reported^69^. A subset of bacterial probes (Supplementary Table 11) that passed our criteria of probe selection (see SMI section) was included for analysis. Morphology markers, including Alexa Fluor 488-SYTO13 (1:10,000; Bruker Spatial Biology, cat. no. 121300303, lot no. 2566222) and Alexa Fluor 594-CD3 (1:100; Bruker Spatial Biology, cat. no. NBP2-54392AF594, lot no. D133856), were used to detect nuclei and TILs, respectively. ROIs (300 µm in diameter) were selected from areas with high-TIL and low-TIL areas in brain tumors (2–6 ROIs per sample). Oligo barcodes were cleaved using the GeoMx digital spatial profiler (Bruker Spatial Biology). RNA and protein barcodes were sequenced at a 2:1 concentration ratio, at 300× using the NovaSeq 6000 sequencer (Illumina) on the S2 flow cell with 27-bp paired-end reads.

#### Statistical analysis of DSP data

Raw counts were quality checked, and ROIs with at least 80% trimmed, stitched and aligned reads and more than 50% sequencing saturation and a minimum of 1,000 reads were selected for analysis. The negative probes were quality controlled, and outlier probes were removed, as recommended by Bruker Spatial Biology. Pan-bacterial 16S probe sequences were similar to those described for CosMx but tagged for GeoMx assay. Geometric mean of the normalized 16S counts from these probes was used to determine low-16S and high-16S regions as first and fourth quartiles, respectively.

Limit of quantification (LOQ) was calculated based on geometric means of the negative probes using the following formula: LOQ = geomean (negative probe) × geoSD (negative probe)^*n*^. For both glioma and BrM, two geometric standard deviations above the geometric mean was used. ROIs with less than 10% gene detection rate and genes detected in less than 10% of the ROIs were excluded, resulting in the selection of 91 of 92 ROIs and 13,093 genes for glioma and 76 of 90 ROIs and 13,990 genes for BrM. To compensate for signal-to-noise variability among samples, we conducted background subtraction followed by Q3 normalization or background normalization for WTA and 16S counts, respectively. For analyses that required log_2_ transformation, data were thresholded by replacing values <1 with the maximum normalized value <1; otherwise, unthresholded data were used.

Using the same ROIs selected for RNA analysis, proteins that had a signal-to-noise ratio of more than 2 in at least 10% of the ROIs were selected for analysis (BrM 260/580 probes; glioma 333/580 probes). On the selected proteins, background normalization was conducted using IgGs.

PCA was performed on WTA log_2_-normalized counts using prcomp with centering and scaling; group differences by categorical 16S status (16S-high or 16S-low regions) were assessed with PERMANOVA (adonis, vegan R package).

Differential protein and transcript abundance in 16S-high and 16S-low regions was compared using an LMM with bacterial status as a fixed effect and patient ID as a random intercept to account for repeated measures implemented using the mixedModelDE function in the GeoMxTools R package. The Benjamini–Hochberg post hoc test was used for multiple hypothesis testing correction. For glioma, LMM was also controlled for IDH and recurrence, with two ROIs from P10 lacking these annotations omitted from the differential expression analysis. Proteins and transcripts that had an adjusted *P* < 0.05 and log_2_FC > 0.58 were considered significant and are presented in Supplementary Table 5 and volcano plots (Extended Data Fig. 4c,d). Proteins and transcripts with immune and metabolism relevance and log_2_FC > 0.58 were selected for presentation using bubble plots (Fig. 4c,d and Extended Data Fig. 4e,f). The association of 16S signal with general inflammatory processes in the tumor was conducted using a reference signature, including CD68, CD14, CD44, IL-6R, MMP9, STAT3 and C-reactive protein.

### Quantification and statistical analysis

Analysis of microbiome data for differential abundance was performed using MaAsLin2 (version 1.20) and ANCOM-BC (version 2.8.1.) as described. Spatial transcriptomics and proteomic data were normalized as noted and analyzed for gene enrichment using a generalized LMM (SMI) or an LMM (DSP and SMI pathway analysis) and the Benjamini–Hochberg procedure for multiple hypothesis testing correction. Adjusted *P* values less than 0.05 were considered significant. PCA group differences were assessed by PERMANOVA. Correlation of tumor characteristics, clinical outcome and presence or absence of intratumoral 16S signal was analyzed using Pearson’s chi-squared test or Wilcoxon rank-sum exact test. *P* values less than 0.05 were considered significant.

### Reporting summary

Further information on research design is available in the Nature Portfolio Reporting Summary linked to this article.

## Online content

Any methods, additional references, Nature Portfolio reporting summaries, source data, extended data, supplementary information, acknowledgements, peer review information; details of author contributions and competing interests; and statements of data and code availability are available at 10.1038/s41591-025-03957-4.

## Supplementary information

Reporting Summary

**Supplementary Table 1** Summary of methodologies applied to individual samples and corresponding results on detection of bacterial signal**Supplementary Table 2** Description of samples included in TMAs constructed from patients with glioma and BrM for DSP and SMI**Supplementary Table 3** Description of tissue (tumor and non-cancerous brain tissue), stool, saliva and cheek swab samples from all cohorts included for sequencing in this study with associated demographic and clinical information**Supplementary Table 4** Bacterial taxa identified through shotgun metagenomic sequencing in tumor samples and the associated genome coverage information**Supplementary Table 5** Differentially enriched proteins and transcripts in bacterial 16S-high and 16S-low regions of interest in BrM and glioma samples**Supplementary Table 6** Differentially enriched transcripts in 16S-positive cells and neighborhoods in BrM and glioma samples**Supplementary Table 7** Differentially abundant oral and gut bacterial taxa in patients with brain tumor compared to healthy individuals (HMP)**Supplementary Table 8** Bacterial taxa identified through shotgun metagenomic sequencing in matched brain metastasis and primary tumor samples**Supplementary Table 9** Differentially abundant oral and gut bacterial taxa in patients with brain tumor with or without progression post-surgery**Supplementary Table 10** Correlation of gut, oral and intratumoral bacterial signatures with clinical characteristics and outcome**Supplementary Table 11** Bacterial probe sequences for SMI and DSP**Supplementary Table 12** Curated gene sets used for custom pathway analysis in small molecular imaging cell and neighborhood analyses

Supplementary Video 1 Three-dimensional reconstruction of representative tumor cells with high-confidence and low-confidence intracellular 16S signal

Supplementary Video 2 Three-dimensional reconstruction of representative tumor cells harboring genus-specific bacterial signal

## Data Availability

Deidentified patient and microbial data have been deposited in the NCBI Sequence Read Archive, and raw sequence data from digital spatial profiling and spatial molecular imaging have been deposited in the Gene Expression Omnibus (GEO); all data are publicly available under accession number PRJNA1023304. Deidentified FISH and IHC microscopy images reported in this study have been deposited to figshare and are publicly available at https://figshare.com/s/de76f67f2c6dc7f3dbbe.

## Code availability

All original code used to analyze the sequencing and spatial profiling data is provided at https://github.com/mda-primetr/gmorad.braintumors.
