# Supplementary material for: Microbial signals in primary and metastatic brain tumors
Source: Nat Med. 2025 Nov 14;31(11):3675–88. doi: 10.1038/s41591-025-03957-4 (PMC12618227; doi:10.1038/s41591-025-03957-4)
Supplement: Supplementary file 1 — Reporting Summary [file 41591_2025_3957_MOESM1_ESM.pdf]

nature portfolio

Corresponding author(s): Golnaz Morad; Jennifer A Wargo

Last updated by author(s): Aug 8, 2025

Reporting Summary

Nature Portfolio wishes to improve the reproducibility of the work that we publish. This form provides structure for consistency and transparency in reporting. For further information on Nature Portfolio policies, see our Editorial Policies and the Editorial Policy Checklist.

Statistics

For all statistical analyses, confirm that the following items are present in the figure legend, table legend, main text, or Methods section.

n/a	Confirmed	
☐	☒	The exact sample size (*n*) for each experimental group/condition, given as a discrete number and unit of measurement
☐	☒	A statement on whether measurements were taken from distinct samples or whether the same sample was measured repeatedly
☐	☒	The statistical test(s) used AND whether they are one- or two-sided<br>*Only common tests should be described solely by name; describe more complex techniques in the Methods section.*
☐	☒	A description of all covariates tested
☐	☒	A description of any assumptions or corrections, such as tests of normality and adjustment for multiple comparisons
☐	☒	A full description of the statistical parameters including central tendency (e.g. means) or other basic estimates (e.g. regression coefficient) AND variation (e.g. standard deviation) or associated estimates of uncertainty (e.g. confidence intervals)
☐	☒	For null hypothesis testing, the test statistic (e.g. *F*, *t*, *r*) with confidence intervals, effect sizes, degrees of freedom and *P* value noted<br>*Give P values as exact values whenever suitable.*
☒	☐	For Bayesian analysis, information on the choice of priors and Markov chain Monte Carlo settings
☒	☐	For hierarchical and complex designs, identification of the appropriate level for tests and full reporting of outcomes
☐	☒	Estimates of effect sizes (e.g. Cohen's *d*, Pearson's *r*), indicating how they were calculated

Our web collection on statistics for biologists contains articles on many of the points above.

Software and code

Policy information about availability of computer code

Data collection

Digital spatial profiling data was collected using the GeoMx_NGS_Pipeline_3.1.1.6 software (Bruker). Spatial molecular imaging data was collected using CosMx software v 1.0.0 (Bruker).

Data analysis

Tumor 16S rRNA sequencing data were analyzed using DADA2 package (v1.26), phyloseq package (v1.50.0), Changepoint R package (v2.3), SCRuB (v.0.0.1). Metagenomic shotgun sequencing data were analyzed using BBMap (v.38.84), MetaPhlAn package (version 4.1.1.), CHOCOPhlAn database (mpa_vJan21_CHOCOPhlAnSGB_202103). Differential abundance of bacterial taxa in saliva, cheek swab, and stool sample were evaluated by Microbiome Multivariable Association with Linear Models (MaAsLin2, v1.20.0) and Analysis of Compositions of Microbiomes with Bias Correction (ANCOM-BC vs. ANCOM-BC2 functions within package v2.8.1. To identify confounding factors, the distribution of different clinical criteria was evaluated by Pearson’s Chi-squared test or Wilcoxon rank sum exact test, and the differential abundance analyses were controlled for criteria with disproportionate distribution. The ggplot2 (v3.5.2) and ggalluvial (v0.12.5) packages were used for data visualization.
For spatial molecular imaging analysis: Semi-supervised cell typing was conducted using a negative binomial model with the InSituType package (https://github.com/Nanostring-Biostats/InSituType), using the default settings (Danaher et al., 2022, doi: https://doi.org/10.1101/2022.10.19.512902). Differential expression was analyzed using the R package "nebula." A NanoString-developed custom plugin for the image software Napari (v0.4.17) and the Plotly package (v5.21.0) in a Python (v3.9.6) environment were used for data visualization. Digital Spatial Profiling data were analyzed and visualized using GeomxTools_3.6.2, NanoStringNCTools_1.10.1, ggplot2_3.5.0, tidyverse_2.0.0, circlize_0.4.16, GeoMxWorkflows_1.8.0, and ComplexHeatmap_2.18.0.

For manuscripts utilizing custom algorithms or software that are central to the research but not yet described in published literature, software must be made available to editors and reviewers. We strongly encourage code deposition in a community repository (e.g. GitHub). See the Nature Portfolio guidelines for submitting code & software for further information.

Data

Policy information about availability of data

All manuscripts must include a data availability statement. This statement should provide the following information, where applicable:

- Accession codes, unique identifiers, or web links for publicly available datasets
- A description of any restrictions on data availability
- For clinical datasets or third party data, please ensure that the statement adheres to our policy

Data availability: Deidentified patient and microbial data have been deposited in the NCBI Sequence Read Archive (SRA) and raw sequence data from digital spatial profiling and spatial molecular imaging have been deposited in Gene Expression Omnibus (GEO); all data are publicly available under accession number PRJNA1023304 at https://www.ncbi.nlm.nih.gov/bioproject/PRJNA1023304. Deidentified FISH AND IHC microscopy images reported in this study have been deposited to Figshare and are publicly available at https://figshare.com/s/de76f67f2c6dc7f3dbbe. All inquiries regarding this study can be directed to the corresponding authors (gmorad@mdanderson.org, jwargo@mdanderson.org), with an expected response within 2 weeks.
Code availability: All original code used to analyze the sequencing and spatial profiling data has been provided at: https://github.com/mda-primetr/gmorad.braintumors.

Research involving human participants, their data, or biological material

Policy information about studies with human participants or human data. See also policy information about sex, gender (identity/presentation), and sexual orientation and race, ethnicity and racism.

Reporting on sex and gender	Sex and gender were not considered as inclusion or exclusion criteria during patient recruitment or sample selection. Sex information was collected based on patients electronic medical record. For all clinical analyses (correlation with clinical characteristics and progression), sex (self-report) was assessed for disproportionate distribution and was found not to be a confounding factor (Supplementary Table 8). Gender information was not available in medical charts and therefore, not recorded for this study.
Reporting on race, ethnicity, or other socially relevant groupings	Race, ethnicity, and other social groupings were not considered as inclusion or exclusion criteria during patient recruitment, sample selection or analysis. Race was identified based on patients electronic medical record. For all clinical analyses (correlation with clinical characteristics and progression), age and race were assessed for disproportionate distribution. In assessment of the correlation of the gut and oral microbiome with post-resection progression in glioma, age was found to be a confounding factor, and was incorporated in the analysis.
Population characteristics	Demographic information including age, sex, and race as well as clinical and sample-related information are reported in Supplementary Table 1 and 3.
Recruitment	MD Anderson Cancer Center: Patients with primary and metastatic brain tumors undergoing surgical resection of brain lesions were enrolled in this study. University of Texas Health Science Center at Houston: Non-cancerous patients were enrolled in this study as part of resective epilepsy procedure for medically refractory epilepsy. Patients with metastatic brain tumors were prospectively enrolled to collect tumor samples for microbiome analysis. All patients provided informed written consent prior to participation in this study.
Ethics oversight	This study was IRB approved at MD Anderson Cancer Center and University of Texas Health Science Center at Houston.

Note that full information on the approval of the study protocol must also be provided in the manuscript.

Field-specific reporting

Please select the one below that is the best fit for your research. If you are not sure, read the appropriate sections before making your selection.

☒ Life sciences ☐ Behavioural & social sciences ☐ Ecological, evolutionary & environmental sciences

For a reference copy of the document with all sections, see nature.com/documents/nr-reporting-summary-flat.pdf

Life sciences study design

All studies must disclose on these points even when the disclosure is negative.

Sample size	Sample size for each experiment is indicated in the figure and/or figure legend. No statistical method was used to predetermine sample size.
Data exclusions	Spatial molecular imaging: excluded cells in which 16S bacterial signals were positioned close to the cell membrane. Cells with fewer than 20 total transcripts were also excluded from the analysis.<br>16S rRNA sequencing: samples that did not yield any sequencing reads were excluded from analysis.<br>Digital spatial profiling: ROIs with less than 10% gene detection rate and genes detected in less than 10% of the ROIs were excluded.
Replication	For digital spatial profiling and spatial molecular imaging, multiple regions of interest and fields of view were profiled on the same tissue microarray.

Randomization	No experimental groups were assigned in this study.
Blinding	No experimental groups were assigned in this study. Clinical information was collected by a neurosurgeon who was blind to microbial analyses.

Reporting for specific materials, systems and methods

We require information from authors about some types of materials, experimental systems and methods used in many studies. Here, indicate whether each material, system or method listed is relevant to your study. If you are not sure if a list item applies to your research, read the appropriate section before selecting a response.

Materials & experimental systems

n/a	Involved in the study	
☐	☒	Antibodies
☒	☐	Eukaryotic cell lines
☒	☐	Palaeontology and archaeology
☒	☐	Animals and other organisms
☐	☒	Clinical data
☒	☐	Dual use research of concern
☒	☐	Plants

Methods

n/a	Involved in the study	
☒	☐	ChIP-seq
☒	☐	Flow cytometry
☒	☐	MRI-based neuroimaging

Antibodies

Antibodies used	LPS (1:500; Abcam, #ab8467; lot# GR3442000- 1), CD8 (1:200; Novus Biologicals, # NBP2-29475), CD4 (1:1000; Abcam, # 133616), Granzyme B (1:100; Cell Signaling, # 46890), FoxP3 (1:50; Cell Signaling, # 98377), CD16 (1:12,000; Invitrogen, # Pa5-80622), CD56 (1:50; Cell Signaling, # 99746), IBA1 (1:8000; Abcam #178847), CD163 (1:500; Abcam, #182422), CD206 (1:4000; Abcam, #64693), Arginase 1 (1:4500; Invitrogen, # PA5-85267), CD11b (1:6000; Abcam, #133357), CD11c (1:400; Cell Signaling, # 45581), Opal 7 color kit (Akoya Biosciences, # NEL871001KT), SYTO13 (1:10,000; Thermofisher Scientific # S7575, lot # 2566222), CD3 (1:100; Novus Biologicals, # NBP2-54392AF594, lot # D133856), Histone H3 (NanoString Technologies, CosMx Human 6k Discovery panel, # 121500041, lot# 0519127), GFAP (NanoString Technologies, CosMx Human 6k Discovery panel, # 121500041, lot# 0519126), PanCK/CD45 (NanoString Technologies, CosMx Human 6k Discovery panel, # 121500041, lot# LN0519123), and CD298/B2M (NanoString Technologies, CosMx Human 6k Discovery panel, # 121500041, lot# LN0519122).
Validation	All antibodies used in this study are commercially available and have been validated by the manufacturer and/or previous publications.

Clinical data

Policy information about clinical studies
All manuscripts should comply with the ICMJE guidelines for publication of clinical research and a completed CONSORT checklist must be included with all submissions.

Clinical trial registration	n/a
Study protocol	MD Anderson Cancer Center: All samples were collected under the institutional approved IRB protocol (2012-4041); University of Texas Health Science Center at Houston: All samples were collected under the institutional approved IRB protocol (HSC-MS-0967). Study protocols have been provided in Supplementary Information.
Data collection	Samples were collected from patients with primary and metastatic brain tumors undergoing surgical resection of brain lesions. Samples from non-cancerous patients were collected as part of resective epilepsy procedure for medically refractory epilepsy. Samples were collected from a total of 145 patients at MD Anderson Cancer Center and University of Texas Health Science Center at Houston from 2019-2023. Demographic information including age, sex, and race as well as clinical and sample-related information are reported in Supplementary Information.
Outcomes	n/a, observational study.

Plants

Seed stocks	n/a
Novel plant genotypes	n/a
Authentication	n/a
